# Advanced Carbon–Nickel Sulfide Hybrid Nanostructures: Extending the Limits of Battery-Type Electrodes for Redox-Based Supercapacitor Applications

**DOI:** 10.1021/acsami.1c03053

**Published:** 2021-04-21

**Authors:** Neelakandan M. Santhosh, Kush K. Upadhyay, Petra Stražar, Gregor Filipič, Janez Zavašnik, André Mão de Ferro, Rui Pedro Silva, Elena Tatarova, Maria de Fátima Montemor, Uroš Cvelbar

**Affiliations:** † Department of Gaseous Electronics, Jožef Stefan Institute, Jamova Cesta 39, Ljubljana SI-1000, Slovenia; ‡ Jožef Stefan International Postgraduate School, Jamova Cesta 39, Ljubljana SI-1000, Slovenia; § Charge2C-NewCap, Av. José Francisco Guerreiro, No 28 Paiã Park, Armazém A2.12, Pontinha, Odivelas 1675-078, Portugal; ∥ Centro de Química Estrutural-CQE, Departamento de Engenharia Química, Instituto Superior Técnico, Universidade de Lisboa, Lisboa 1049-001, Portugal; ⊥ Instituto de Plasmas e Fusão Nuclear, Instituto Superior Técnico, Universidade de Lisboa, Lisboa 1049, Portugal

**Keywords:** nickel sulfide, heazlewoodite Ni_3_S_2_, vertical carbon nanostructure, redox-based supercapacitor, binder-free, plasma synthesis

## Abstract

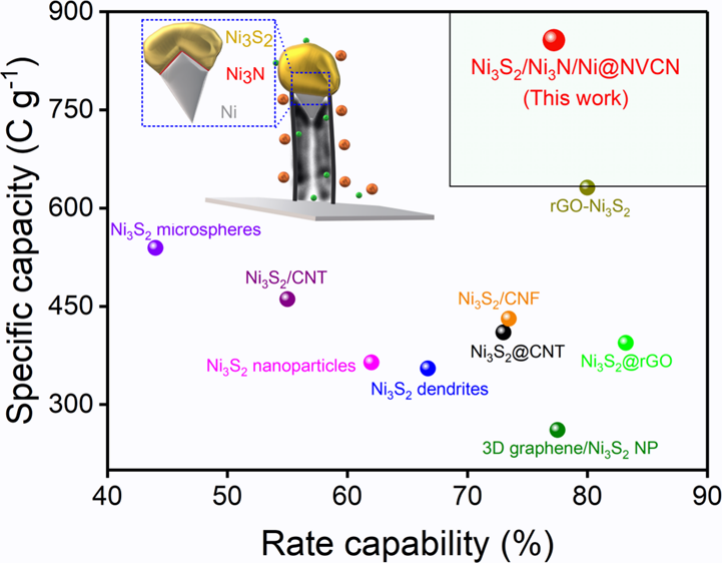

Transition-metal sulfides combined with conductive carbon nanostructures are considered promising electrode materials for redox-based supercapacitors due to their high specific capacity. However, the low rate capability of these electrodes, still considered “battery-type” electrodes, presents an obstacle for general use. In this work, we demonstrate a successful and fast fabrication process of metal sulfide–carbon nanostructures ideal for charge-storage electrodes with ultra-high capacity and outstanding rate capability. The novel hybrid binder-free electrode material consists of a vertically aligned carbon nanotube (VCN), terminated by a nanosized single-crystal metallic Ni grain; Ni is covered by a nickel nitride (Ni_3_N) interlayer and topped by trinickel disulfide (Ni_3_S_2_, heazlewoodite). Thus, the electrode is formed by a Ni_3_S_2_/Ni_3_N/Ni@NVCN architecture with a unique broccoli-like morphology. Electrochemical measurements show that these hybrid binder-free electrodes exhibit one of the best electrochemical performances compared to the other reported Ni_3_S_2_-based electrodes, evidencing an ultra-high specific capacity (856.3 C g^–1^ at 3 A g^–1^), outstanding rate capability (77.2% retention at 13 A g^–1^), and excellent cycling stability (83% retention after 4000 cycles at 13 A g^–1^). The remarkable electrochemical performance of the binder-free Ni_3_S_2_/Ni_3_N/Ni@NVCN electrodes is a significant step forward, improving rate capability and capacity for redox-based supercapacitor applications.

## Introduction

The increasing demand for power backup solutions and the use of renewable energy resources for fulfilling consumer requirements have prompted the development of advanced electrochemical energy storage devices such as batteries and supercapacitors.^1−3^ Supercapacitors have many practical applications and have attracted research attention due to their high power density, long cycle life, and fast charge–discharge rate properties.^4,5^ However, large-scale applications of supercapacitors are still limited due to their low energy density.^6,7^ To overcome these shortcomings, research has focused on the exploration of new electrochemically redox-active materials with higher specific capacity and new electrolytes to boost supercapacitor performance.^8−10^ Most current commercial electrolytes are organic and many are toxic, flammable, of high cost, and generally not environmentally friendly.^11^ Hence, efforts are being made to develop hybrid high-performing electrode materials able to work in aqueous electrolytes.^12,13^

Materials for redox-based supercapacitor electrodes are expected to meet three criteria: first, the high electrical conductivity of the electrode material for the fast charge carrier transport to the electrode interface from the electrolyte; second, high surface area because the capacity scales with the interfacial area; and third, excellent electrode electrochemical stability to avoid unwanted reactions with the electrolyte and decreased stability in long-term cycling. Thus, electrode materials need to be designed to withstand long-term cycling with enhanced electrochemical activity. In this aspect, novel electrode materials for supercapacitors can be classified according to their charge-storage process, which can be either non-Faradaic or Faradaic.^14,15^ In the former type, charge storage is induced by charge accumulation at the surface of the electrodes. In the second case, charge storage is achieved by Faradaic electron-charge transfer with fast and quasi-reversible redox processes that involve intercalation and electrosorption. Compared to non-Faradaic double-layer electrodes, the Faradaic type has the potential to deliver much higher capacity and energy density due to reversible redox reactions that are the main contributors to the charge storage.^16^ In the context of Faradaic supercapacitors, charge-storage mechanisms may be classified as pseudocapacitive, displaying a nearly linear galvanostatic charge–discharge (GCD) response, or as battery-like, in which the Faradaic process is explained by the Nernst equation featuring nonlinear GCDs, which can display one or more voltage plateaus.^16,17^ In this regard, materials with a noncapacitive Faradaic-type charge-storage mechanism are known as “battery-type” materials due to similarities with battery electrochemical responses.^18^ In general, materials that exhibit multiple redox valences, such as transition metals (*e.g.*, Ni, Co, Mn, Ti, and Fe) and their compounds (oxides, hydroxides, and sulfides), have been proposed as materials for Faradaic electrodes.^19−22^ Additionally, electrochemical redox-active polymers are also considered as an important type of pseudocapacitive material.^23,24^ It is well known that many metal hydroxides and sulfides display a battery-type response. Compared to other transition-metal compounds, transition-metal sulfides appear particularly suitable for energy storage applications due to their good electrical conductivity, high redox activity, easy preparation, and low cost.^25−28^ Among them, nickel sulfides have the highest capacitance values.^29^ Considering the varieties of nickel sulfides, such as NiS_2_,^30,31^ Ni_3_S_2_,^32−34^ Ni_3_S_4_,^28,35^ Ni_7_S_6_,^36^ and Ni_9_S_8_,^37^ superior electrochemical performance has been reported for Ni_3_S_2_-based electrodes. Compared to other nickel sulfide phases, Ni_3_S_2_ structures possess high electrical conductivity, exceptional theoretical capacity, and excellent cyclic performance.^38−40^

However, the use of nickel sulfide-based battery-type electrode materials for energy storage in commercial applications is still limited due to the poor electrical conductivity and structural stability compared to commercially available carbon-based nanostructures. These factors decrease the stability and rate performance of the electrode materials. An effective strategy to overcome this issue is compositing the nickel sulfide-based electrodes with a highly conductive and stable carbon matrix (*e.g.*, graphene, carbon nanotubes, carbon nanofiber, and carbon nanowall).^41−43^ Direct growth/deposition of electrode materials on the current collector is considered the ideal strategy to provide free-standing hierarchical nanostructure-based electrodes with a large contact area to improve the electrochemical reaction. Additionally, this technique can eliminate resistivity-related issues due to the use of binders during electrochemical testing.^44,45^

A feasible route to overcome the issues related to poor conductivity and other side effects of binders is the direct growth of a carbon matrix on the current collector, followed by compositing it with nickel sulfides. Direct growth of carbon-based nanostructures on different substrates and their processing has been performed in the past using different techniques. Commonly used techniques are chemical vapor deposition, arc-discharge method, electrochemical synthesis, and plasma-enhanced chemical vapor deposition (PECVD).^46−48^ Compared to other techniques, the PECVD technique allows the fastest controllable growth of various carbon nanostructures with different morphologies and orientations.^47,49^ The tip-growth mechanism is a well-known catalyst-assisted plasma deposition technique for the synthesis of vertical carbon nanotubes, resulting in each nanotube being terminated with the catalyst nanoparticle, which was also successfully demonstrated for Ni.^50−52^ Combined with the possibility of post-synthesis alterations of the terminating Ni into nickel sulfide, the technique appears to be a highly promising approach to fabricate complex multiphase electrodes for energy storage applications. These nickel sulfide-carbon-based structures possess unique electrochemical properties compared to nickel sulfide and carbon when used individually as active materials. As a result, the composite provides a higher capacity, better cycling stability, and good rate capability, making it suitable for assembling high-capacity redox-based supercapacitors.

The electronic properties of carbon nanostructures can be tailored by chemical doping either by adsorption of foreign molecules on the carbon lattice or by substitutional doping by nitrogen, oxygen, or boron, which can either donate or withdraw free electrons.^53,54^ Among the varieties of heteroatoms, nitrogen is considered as a promising dopant since the presence of nitrogen also facilitates fast surface redox reactions, further improving the pseudocapacitive properties of the electrode materials.^55^ Thus, the presence of additional nitrogen in the above-mentioned nickel sulfide-carbon nanostructure-based electrodes is expected to enhance electrochemical performance significantly.

This work proposes a fast and facile approach for the direct fabrication of a hybrid electrode material based on trinickel disulfide (Ni_3_S_2_) and vertically aligned carbon nanotubes (VCNs). The VCN structures are synthesized on Ni foil using an inductively coupled radio-frequency PECVD system. The VCN structures are terminated with a single-crystal metallic Ni nanoparticle at the tip, forming Ni@VCN. To improve the electronic properties of the carbon backbone, Ni@VCN structures are subjected to N-doping. Along with the N-doped VCN (NVCN), the exposed part of Ni in Ni@VCN is covered with an atomically thin layer of nickel nitride (Ni_3_N), resulting in a Ni_3_N/Ni@NVCN architecture. These structures are subjected to low-temperature annealing (125 °C) in the presence of H_2_S gas to achieve the transformation to NiS. In the case of undoped Ni@VCN, the resulting Ni_3_S_2_ is polycrystalline with a broccoli-like morphology, while in the case of the N-doped samples with an intermediate Ni_3_N layer, the process results in single-crystal Ni_3_S_2_ on top of Ni_3_N/Ni@NVCN. The fabricated binder-free electrodes exhibit a Faradaic “battery-like” electrochemical response. Ni_3_S_2_/Ni_3_N/Ni@NVCN electrodes show one of the highest discharge capacities among Ni_3_S_2_-based electrodes. Until now, one of the main challenges of translating these Faradaic materials into supercapacitor electrode applications has been poor rate capability. However, the fabricated Ni_3_S_2_/Ni_3_N/Ni@NVCN retains 78% of the initial capacity at a higher current density of 13 A g^–1^. The unique broccoli-like morphology of the hybrid vertical structures, the formation of defects in the crystalline structures during electrochemical reactions, and the direct contact between the current collector and active materials all contribute to boosting the electrochemical performance, capacity, and rate capability of the hybrid electrodes. Thus, this hybrid material is extremely promising as a basis for electrodes in supercapacitor applications.

## Results and Discussion

### Nanostructure and Morphology Characterization

**Figure 1:**
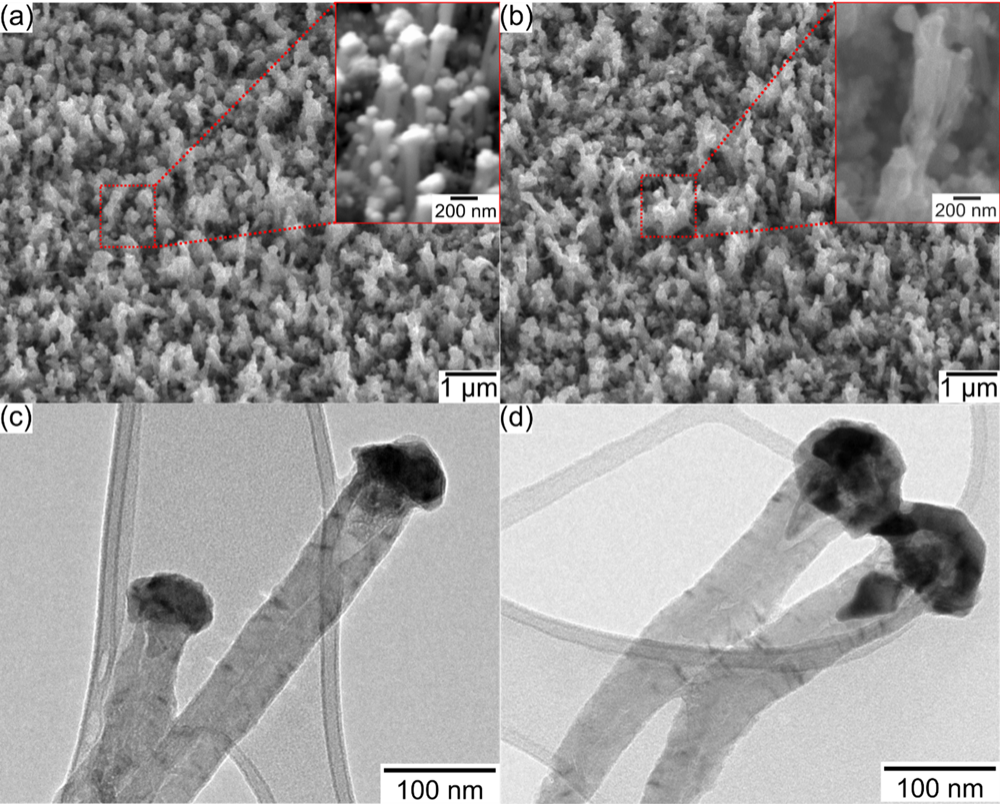
Secondary electron (SE) SEM images of nickel sulfide/carbon composites before and after N-doping. Tilted view of (a) nickel sulfide/carbon nanostructures and (b) nickel sulfide/N-doped carbon nanostructures. TEM micrograph of (c) nickel sulfide/carbon nanostructures and (d) nickel sulfide/N-doped carbon nanostructures.

The initial carbon nanostructures were deposited on Ni foil using a catalyst-assisted plasma-deposition technique (PECVD) reported similarly elsewhere^50,51^ and were grown in the form of VCNs, capped by a single-crystal Ni nanoparticle. N-doping did not alter the samples significantly, and the VCN and NVCN both had an approximate height of ∼1 μm (Figure S1a,b). Then, the samples were subjected to sulfur treatment and analyzed by scanning electron microscopy (SEM). The surface morphology of the uniformly grown and densely packed sulfur-treated VCN and NVCN is presented in Figure 1a,b. When comparing the VCN and NVCN, the tips of the individual tubes became swollen after sulfur treatment, while the dimension and orientation of the nanotubes remained intact; apart from that, no other differences were observed. Next, the structure and morphology of sulfur-treated VCN and NVCN were analyzed at the nanoscale by transmission electron microscopy (TEM; Figure 1c,d). In both cases, the metal tip of the VCN changed and expanded, corresponding to broccoli-like polycrystalline NiS for the VCN and to a single-crystal NiS for the NVCN.

**Figure 2:**
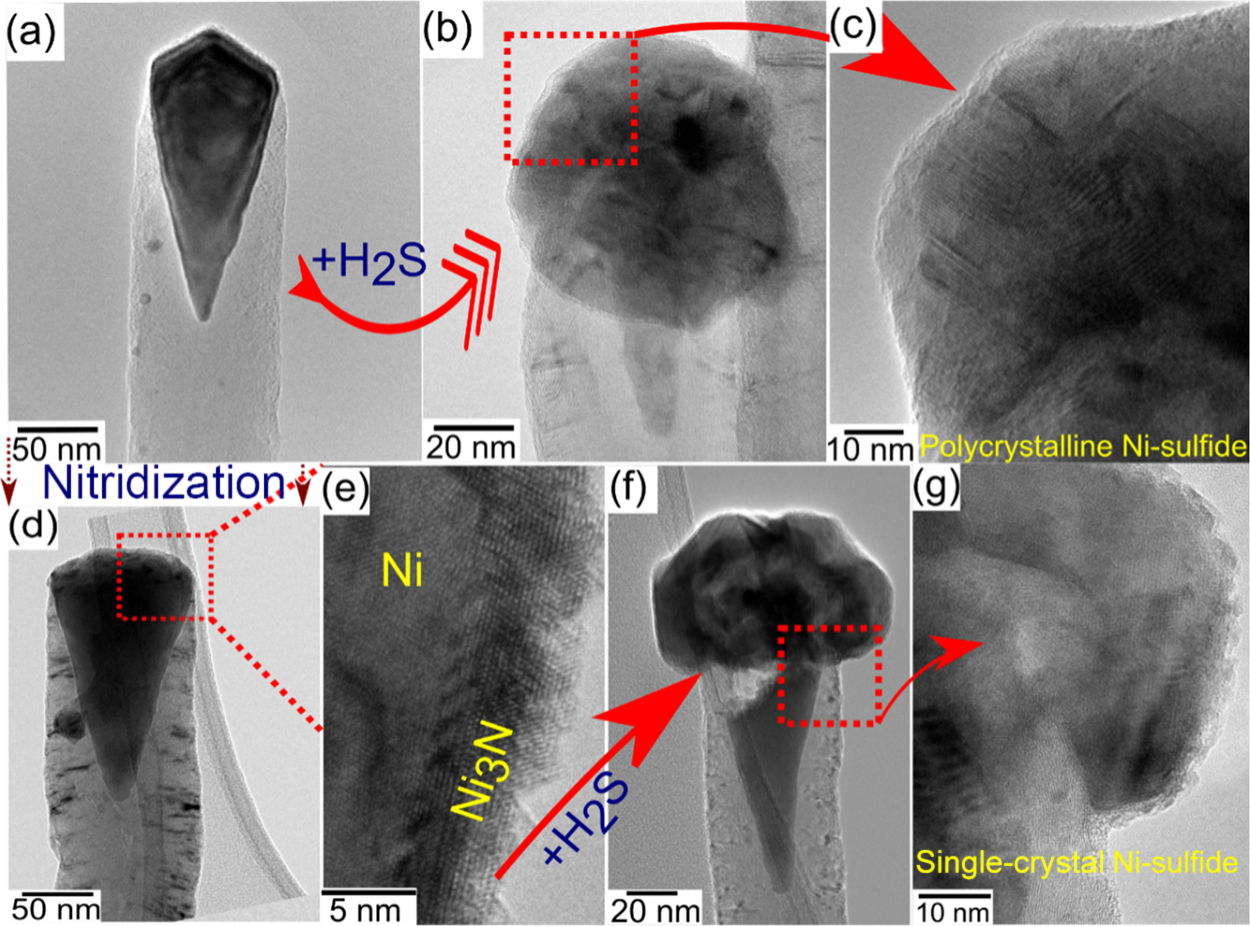
TEM micrograph of (a) Ni@VCN, (b) NiS grown at the Ni@VCN, (c) HR-TEM micrograph of polycrystalline nickel sulfide formed on Ni@VCN, (d) Ni_3_N/Ni@NVCN, (e) HR-TEM micrograph of Ni_3_N/Ni, (f) NiS grown at the top of Ni_3_N/Ni@NVCN, and (g) HR-TEM micrograph of single-crystalline nickel sulfide formed on Ni_3_N/Ni@NVCN.

To understand the formation and transformation of these sulfur phases, TEM and high-resolution TEM (HR-TEM) analyses were conducted on VCN and NVCN before and after sulfur treatment (Figure 2). The initial VCN was a multiwalled carbon nanotube, with an average diameter of 50 nm, terminated by a faceted, single-crystal metallic Ni nanoparticle (Figure 2a), forming a Ni@VCN structure. The NVCN showed similar features, with an additional nitride layer atop the exposed Ni (Figure 2b,e); the nitride phase formed an epitaxial layer, with a thickness of ∼5 nm corresponding to Ni_3_N (Figure S2). TEM micrographs of sulfur-treated VCN and NVCN are shown in Figure 2b,f, respectively. While neither N nor S treatment modified the carbon structures, and the sulfur neither damaged the vertical alignment, incorporated itself into the carbon backbone, nor deposited as elemental S on the nanostructure, both processes have a significant influence on the terminal Ni monocrystal (Figure S3).

TEM results confirm that in both cases, a successful transformation of the capping single-crystal Ni to NiS was achieved. Sulfur reacted only with the exposed part of the Ni nanoparticle at the VCN tip and was locally converted into NiS, which was observed by SEM as an enlargement of the VCN tip. In both samples, remnants of metallic Ni can still be observed under NiS in the core of the VCN. HR-TEM studies on the hybrid NiS/Ni@VCN and NiS/Ni_3_N/Ni@NVCN structures are presented in Figure 2c,g. In the case of NiS/Ni@VCN structures, NiS is polycrystalline (Figure 2c), with an individual domain size of 20–50 nm, firmly intergrown. In contrast, NiS formed on Ni_3_N/Ni@NVCN is a single crystal of size ∼50–100 nm. During the H_2_S treatment of Ni_3_N/Ni@NVCN, the exposed Ni faces were protected by a thin nitride layer; thus, the sulfur had access to the metallic Ni only where the NVCN carbon layer and nitride layers met, leading to a slow in-diffusion of Ni, resulting in single-crystal NiS formation (Figure 2g). The process of Ni leaching may also explain the formation of a void observed in the Ni core in Ni_3_N/Ni@NVCN, suggesting that no direct contact between Ni and NiS is needed for sulfide formation and that diffusion is only dictated by NVCN. As there are various abundant NiS phases, and their properties depend on the structure, selected area electron diffraction (SAED) was used to determine the NiS phase, identified as heazlewoodite Ni_3_S_2_ (Figure S4a,b) in both structures.^44,56,57^ In addition, Ni_3_N phases were still preserved in the sulfur-treated Ni_3_N/Ni@NVCN structures. Thus, the obtained results confirm that the broccoli-like morphology is a hybrid hierarchical structure of core-shell-type Ni–Ni_3_S_2_ on top of VCN structures (Ni_3_S_2_/Ni@VCN) and Ni_3_N-encapsulated Ni_3_S_2_ nanostructures on NVCN structures (Ni_3_S_2_/Ni_3_N/Ni@NVCN).

### Chemical and Composition Characteristics

**Figure 3:**
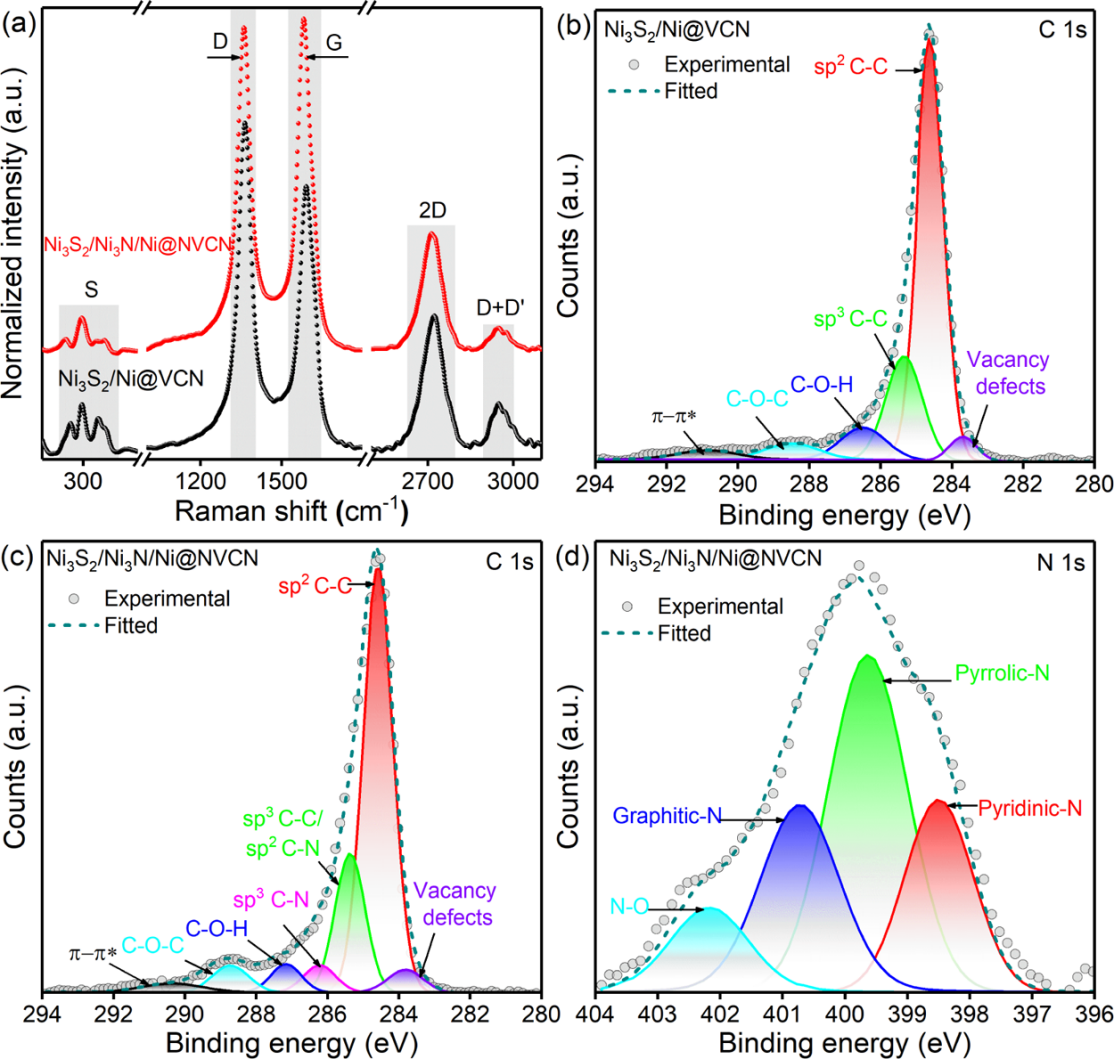
Structural and chemical analysis of the structures. (a) Raman spectra of Ni_3_S_2_/Ni@VCN and Ni_3_S_2_/Ni_3_N/Ni@NVCN, (b) high-resolution C 1s XPS spectrum of Ni_3_S_2_/Ni@VCN, (c) high-resolution C 1s XPS spectrum, and (d) N 1s XPS spectrum of Ni_3_S_2_/Ni_3_N/Ni@NVCN. The XPS spectra are also deconvoluted into different contributing peaks.

Chemical composition and lattice structure analyses of the Ni_3_S_2_/Ni@VCN and Ni_3_S_2_/Ni_3_N/Ni@NVCN hybrid structures were conducted using different spectroscopy techniques. Structural features were characterized by Raman spectroscopy. The spectra of both samples are presented in Figure 3a and feature characteristic peaks of graphene-like structures at 1360 cm^–1^ (D peak), 1581 cm^–1^ (G peak), 2722 cm^–1^ (2D peak), and 2943 cm^–1^ (D + G peak).^58,59^ The presence of D and D + G peaks shows the defect-rich characteristics of the VCN backbone. Typically, the intensity ratio between the D and G bands (*I*_D_/*I*_G_) is used to examine the defect degree of carbon materials. The *I*_D_/I_G_ values for Ni_3_S_2_/Ni@VCN and Ni_3_S_2_/Ni_3_N/Ni@NVCN structures were calculated as 1.2 and 0.98, respectively, which confirmed the presence of defects in the VCN backbone. Apart from the carbon-related peaks, both samples exhibited peaks at 200–400 cm^–1^, which were assigned to peaks from Ni_3_S_2_, as reported in the literature.^57^

Surface components and the chemical composition of the nanostructures were characterized by X-ray photoelectron spectroscopy (XPS) analysis. The presence of C, O, Ni, and S in the nanostructure was confirmed from the XPS survey spectra, which feature peaks at 284.6, 531.8, 850.2, and 162.2 eV, respectively (Figure S5). Besides these, a peak around 400 eV, indicating the presence of N in the structure, was observed in the survey spectrum of Ni_3_S_2_/Ni_3_N/Ni@NVCN.^60,61^ High-resolution C 1s XPS spectra of Ni_3_S_2_/Ni@VCN and Ni_3_S_2_/Ni_3_N/Ni@NVCN are presented in Figure 3b,c. Both spectra display an intense peak at 284.6 eV, which corresponds to sp^2^ C–C bonds. A peak is observed around 285.4 eV in both samples that are assigned to sp^3^ C–C bonds. This peak becomes stronger in Ni_3_S_2_/Ni_3_N/Ni@NVCN structures, which may be due to the presence of sp^2^ C–N after N-doping. Peaks observed at 283.5, 286.7, 288.2, and 290.5 eV can be ascribed to the vacancy defects, the carbon singly bound to oxygen, the carbon in carbonyl groups, and the π–π* shake-up satellite, respectively.^62,63^ Additionally, a peak around 286.2 eV is observed in Ni_3_S_2_/Ni_3_N/Ni@NVCN and attributed to the presence of sp^3^ C–N groups. High-resolution N 1s spectra of the Ni_3_S_2_/Ni_3_N/Ni@NVCN structures have been deconvoluted into four peaks (Figure 3d), namely, pyridinic-N (398.5 eV), pyrrolic-N (399.6 eV), graphitic-N (400.7 eV), and oxides of pyridinic-N (402.2 eV).^64^ Interestingly, there are no peaks from metal nitrides with corresponding lower binding energies (∼397 eV) in N 1s spectra, indicating that the nickel nitride was fully encapsulated by the sulfide.

**Figure 4:**
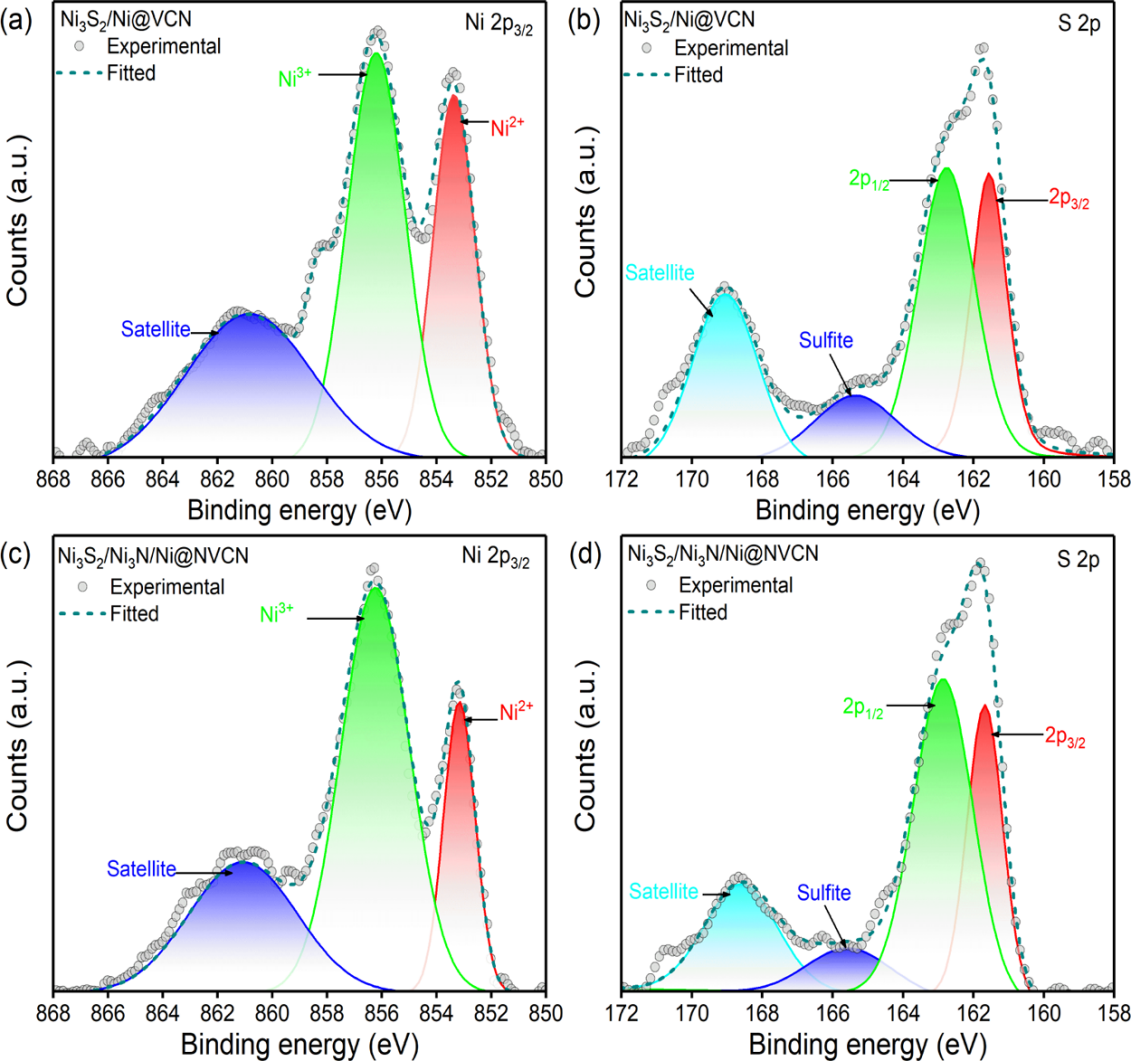
XPS chemical composition of the structures. High-resolution ionizations of (a) Ni 2p and (b) S 2p in Ni_3_S_2_/Ni@VCN. High-resolution ionizations of (c) Ni 2p and (d) S 2p in Ni_3_S_2_/Ni_3_N/Ni@NVCN.

In both samples, Ni 2p_3/2_ ionizations are observed around 853.4, 856.2, and 861.0 eV. These can be ascribed to Ni^2+^, Ni^3+^, and the satellite peak, respectively (Figure 4a,c), which is usually observed in the Ni 2p_3/2_ spectra of Ni_3_S_2_.^65^ The S 2p ionization of both samples features a spin–orbital doublet at 161.7 and 163.0 eV along with a satellite peak at 169.0 eV.^66^ Ionizations are presented in Figure 4b,d. The S 2p ionizations with such doublets asymmetrically broadened to higher binding energies are S 2p characteristics in Ni_3_S_2_ structures.^65,67^ The Ni 2p and S 2p ionization of the N-doped sample and the nondoped sample shows similar spectral features, confirming that the chemical compositions are similar irrespective of the presence of N in Ni_3_S_2_/Ni_3_N/Ni@NVCN.

Detailed information on the peak position and roughly estimated composition ratio (%) from the fitted photoionization of the high-resolution spectra of C 1s, Ni 2p, and S 2p is summarized in Tables S1, S2, and S3, respectively. All the interpretations suggest that sulfurization of Ni@VCN and Ni@NVCN at low temperatures promotes the formation of Ni_3_S_2_-based hybrid structures. Also, the structural and morphological results give an insight into the synthesis of Ni_3_S_2_ with different crystallinities by protecting the exposed part of the Ni nanoparticle with a thin nitride layer. It is a well-known fact that Ni_3_S_2_-based active materials have potential applications for energy storage devices due to their higher electrochemical activity compared to their corresponding oxides.

### Electrochemical Characterization

The combination of Ni_3_S_2_-based active materials with a conductive carbon matrix improves the electrical conductivity of the structure, enabling the easy penetration of electrolyte ions into the active material and facilitating charge transfer during electrochemical processes. Thus, both Ni_3_S_2_/Ni@VCN and Ni_3_S_2_/Ni_3_N/Ni@NVCN materials were tested as electrodes for redox-based supercapacitors. Since the active materials were directly grown on Ni, these architectures were used as binder-free electrodes without any additional processing and considering Ni as the current collector. The electrochemical performance was studied in a three-electrode setup using the fabricated hybrid structures as the working electrode, Pt as the counter electrode, and saturated calomel as the reference electrode.

**Figure 5:**
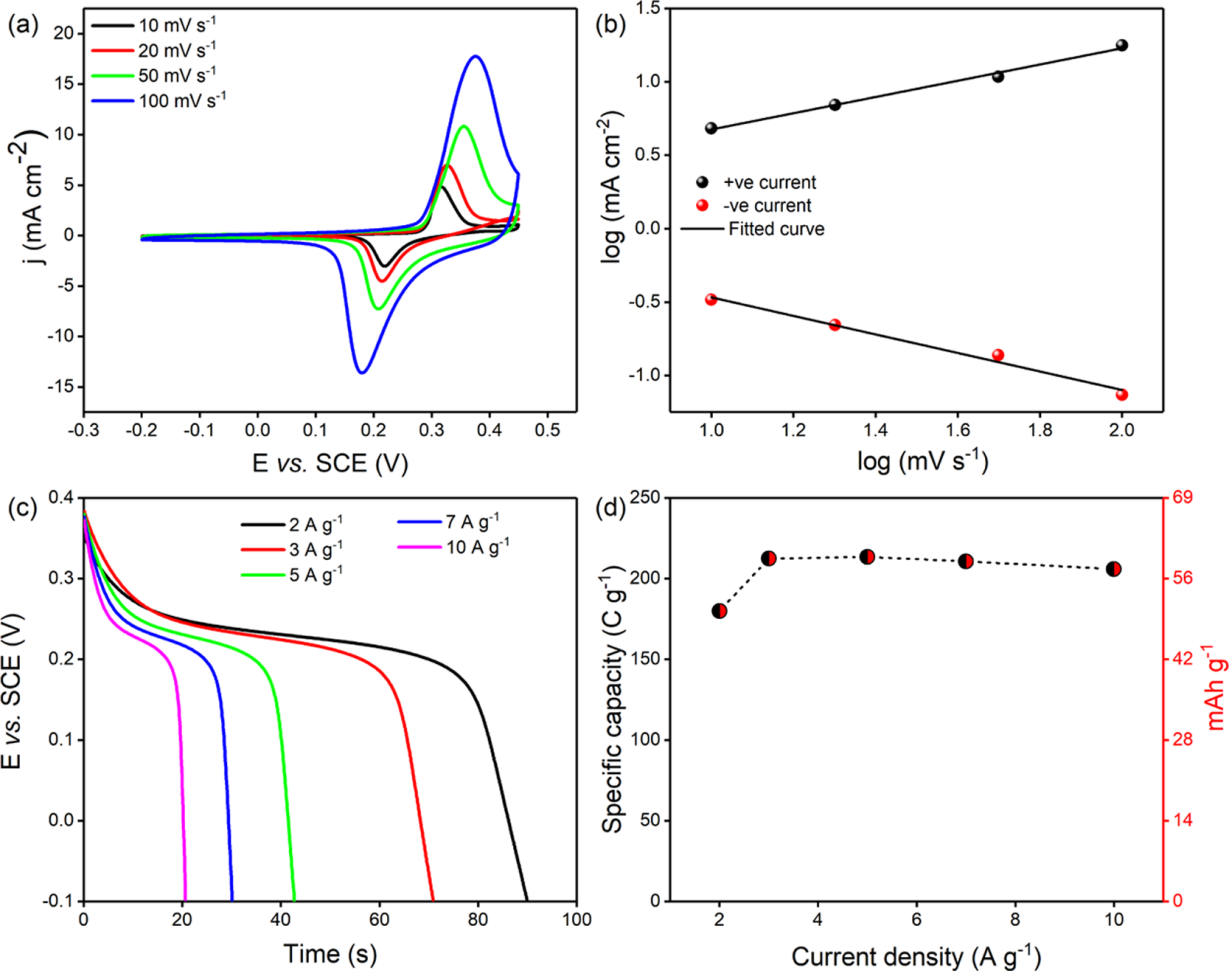
Initial performance of Ni_3_S_2_/Ni@VCN electrodes before cycling. (a) CV at different scan rates, (b) log (current density) *vs* log (scan rate), (c) GCD at different current densities, and (d) rate capability.

Electrochemical measurements of the Ni_3_S_2_/Ni@VCN electrodes are presented in Figure 5. Cyclic voltammetry (CV) curves at different scan rates ranging from 10 to 100 mV s^1^ show well-defined redox peaks and reversible current outputs on the reverse scan (Figure 5a). The anodic peak located around 0.37 V and the cathodic peak around 0.18 V are the evidence for the following proposed electrochemical reaction^44^
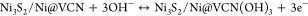
1

Shape retention of the CV curves obtained at different scan rates suggests low internal resistance and quasi-reversibility of the electrode. The reaction kinetics of the electrochemical reaction was deducted from the CV curves using the power-law equation (eq 3).

Figure 5b highlights slopes of 0.55 and 0.63, respectively. This evidences the presence of diffusion-controlled charge-storage mechanisms.^68,69^ Specific capacity and rate capability of the electrodes were investigated by GCD measurements at different current densities, and the results are presented in Figure 5c. GCD curves show a clear plateau, confirming the occurrence of Faradaic-based phenomena. Specific capacity and rate capability performance were calculated using the GCD data using eq 4 and are presented in Figure 5d. The maximum specific capacity of 180 C g^–1^ (50 mA h g^–1^) was obtained at 2 A g^−1^. It is worth noticing that the electrode delivered a specific capacity of 206 C g^−1^ (57.2 mA h g^−1^) at a current density of 10 A g^–1^. Electrodes delivering specific capacity over 100% when the applied current increases fivefold are very unusual and indicate that the electrochemical response was evolving and had not yet stabilized.

**Figure 6:**
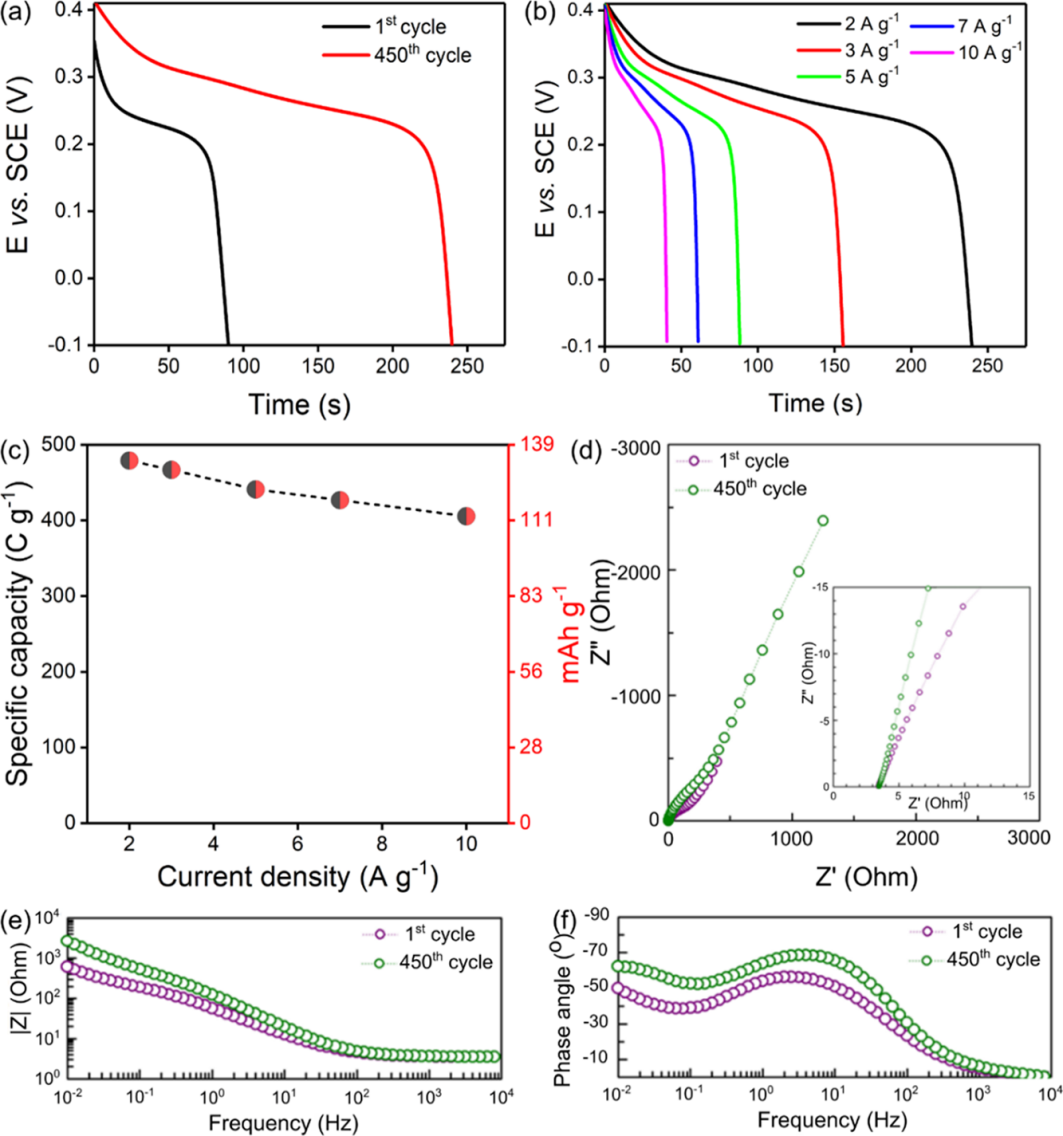
Electrochemical performance of Ni_3_S_2_/Ni@VCN electrodes after stabilization. (a) GCD comparison after the 1st and 450th cycles at 2 A g^–1^, (b) GCD at different current densities after the 450th cycle, and (c) rate capability. EIS plot obtained after the 1st and 450th cycles: (d) Nyquist, (e) magnitude, and (f) phase angle plot.

After this observation, the electrodes were subjected to cycling to study their electrochemical stability. Results evidenced a remarkable improvement in specific capacity after 450 charge–discharge cycles, as observed in the discharge curve comparison plot for the 1st and 450th cycles (Figure 6a). Specific capacity increased by almost 2.6 times the initial value after 450 cycles and attained 479 C g^−1^ at 2 A g^−1^. More importantly, after stabilization, the electrode retained 84% (405.5 C g^–1^) of its initial capacity at a high current density of 10 A g^–1^, as illustrated in Figure 6b,c. These results point to the good electrochemical performance of the directly grown Ni_3_S_2_/Ni@VCN structures.

Electrochemical impedance spectroscopy (EIS) measurements were performed on the as-prepared electrode and after 450 cycles of charge–discharge to understand changes in internal resistance and the charge-transfer process. Nyquist plots are presented in Figure 6d–f. A magnified view of the Nyquist plot (Figure 6d) shows a higher slope for the spectrum taken after 450 cycles toward the imaginary axis that is the result of the enhanced capacitive response. This is confirmed in the phase angle plot (Figure 6f), where after 450 cycles, the electrode attained more negative phase angle values, around −60° in the low-frequency range, and also more negative phase angle values in the high–mid-frequency range. Overall, these results indicate enhanced electrochemical performance on cycling in terms of capacity and, interestingly, cycling seemed to attenuate diffusion limitations observed in the high–mid-frequency region.

**Figure 7:**
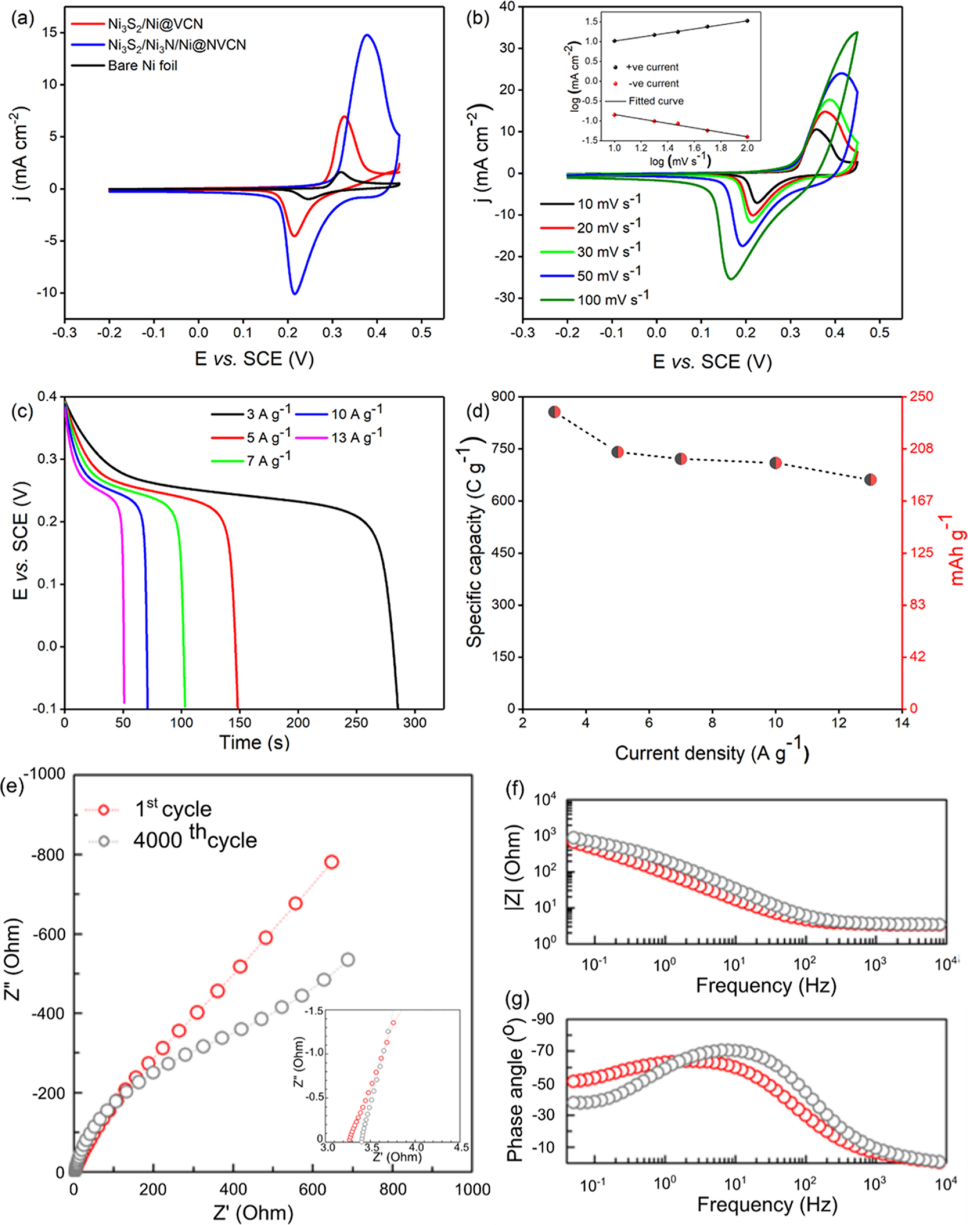
(a) CV comparison of Ni_3_S_2_/Ni@VCN, Ni_3_S_2_/Ni_3_N/Ni@NVCN, and bare Ni foil at 20 mV s^–1^. (b) CV at different scan rates, with the inset showing log (current density) *vs* log (scan rate). (c) GCD at different current densities. (d) Rate capability of the Ni_3_S_2_/Ni_3_N/Ni@NVCN electrode. EIS comparison of Ni_3_S_2_/Ni_3_N/Ni@NVCN after 1st and 4000th cycles. (e) Nyquist plots, magnified view of Nyquist in the high-frequency region (inset), and (f,g) Bode plots.

To investigate the electrochemical responses induced by crystallinity changes and the presence of N in the active material, Ni_3_S_2_/Ni_3_N/Ni@NVCN electrodes were also studied through several electrochemical techniques. CV was performed on Ni_3_S_2_/Ni@VCN, Ni_3_S_2_/Ni_3_N/Ni@NVCN, and bare Ni foil to understand the maximum current gains for different electrodes (Figure 7a). The effect of N-doping in Ni_3_S_2_/Ni_3_N/Ni@NVCN nanostructures is evident from the CV curves, where the electrochemical performance increased almost twofold compared to Ni_3_S_2_/Ni@VCN at a scan rate of 20 mV s^–1^. Bare Ni foil delivered a minimal current response, showing that it has a negligible contribution to the overall current response. Furthermore, CV measurements performed on Ni_3_S_2_/Ni_3_N/Ni@NVCN electrodes at different scan rates (Figure 7b) show clear redox peaks with good reversibility, which can be explained by the following reaction at the interface
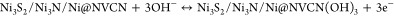
2

Logarithmic analysis of the anodic and cathodic peak current densities *versus* the scan rate based on the CV data (inset of Figure 7b) highlights slopes of 0.5 and 0.56, respectively. This strengthens the argument for the presence of diffusion-controlled charge-storage processes similar to the ones observed for the Ni_3_S_2_/Ni@VCN electrode. Specific capacity and rate capability of the electrode were calculated from GCD measurements at different current densities ranging from 3 to 13 A g^–1^, and results are shown in Figure 7c,d. The Ni_3_S_2_/Ni_3_N/Ni@NVCN electrode delivered a specific capacity of 856.32 C g^−1^ (240.3 mA h g^–1^) at 3 A g^–1^ with a remarkable rate capability, retaining 77.2% of its initial capacity at 13 A g^–1^. It is also worth noting that for the Ni_3_S_2_/Ni_3_N/Ni@NVCN electrode, cycling was not required to reach a stable electrochemical response. Additionally, it was observed that Ni_3_S_2_/Ni@VCN electrodes were more hydrophobic compared to the Ni_3_S_2_/Ni_3_N/Ni@NVCN ones (Figure S6). This could also be a reason for the gradual improvement in the electrochemical performance of the Ni_3_S_2_/Ni@VCN electrodes with cycling, which could be related to the slow and continuous impregnation of electrolytes throughout the active sites. EIS measurements were carried out for the Ni_3_S_2_/Ni_3_N/Ni@NVCN electrode after 4000 cycles, and results were compared to the data obtained on the fresh electrode before cycling (Figure 7e–g). There was a slight increase in the ESR value from 3.3 to 3.4 Ω after cycling, as can be seen in the high-frequency region of the magnified Nyquist plot (inset). The phase angle plot (Figure 7g) has slightly more negative values in the high–mid-frequency range. Impedance studies did not reveal severe detrimental effects on the cycled electrodes from impedance analysis.

**Figure 8:**
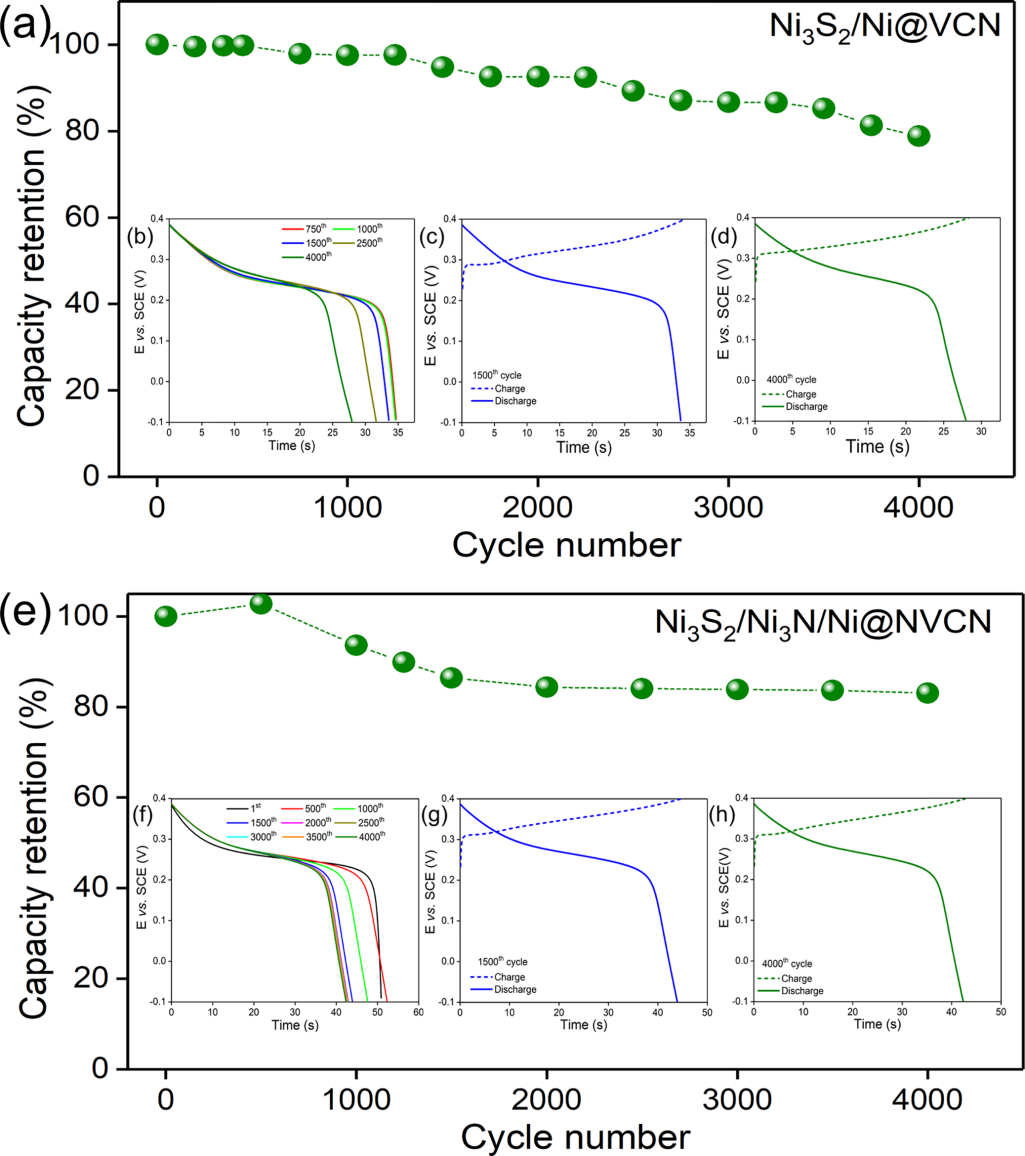
(a) Cyclic stability of Ni_3_S_2_/Ni@VCN up to 4000 cycles at 10 A g^–1^. (b) Discharge plots (c) after 1500th cycle and (d) after 4000th cycle. (e) Cyclic stability of Ni_3_S_2_/Ni_3_N/Ni@NVCN up to 4000 cycles at 13 A g^–1^. (f) Discharge plots (g) after 1500 cycles and (h) after 4000 cycles.

Cyclic stability is an essential feature for a material to be used in redox supercapacitor electrodes. Thus, GCD measurements of the Ni_3_S_2_/Ni@VCN electrode were conducted up to 4000 cycles at 10 A g^–1^, and results are presented in Figure 8a–d.

The Ni_3_S_2_/Ni@VCN electrode retained a specific capacity of 78.8% after 4000 cycles. A decline in performance upon cycling can be seen in Figure 8b, which could be associated with partial morphological distortion and/or material oxidation. Despite reduced stability, the electrode had good Coulombic efficiencies (the ratio of discharge to charge time) of 98.3 and 98.6%, after 1500 and 4000 cycles, respectively (Figure 8c,d). The cyclic stability of the Ni_3_S_2_/Ni_3_N/Ni@NVCN electrode was also measured up to 4000 cycles at a current density of 13 A g^–1^ (Figure 8e). Results showed a slight improvement in capacity up to the initial 500 cycles, and then, the electrode retained 83% of its initial capacity after 4000 GCD cycles, even at higher current densities compared to the Ni_3_S_2_/Ni@VCN electrode. The evolution of the discharge profile upon cycling is illustrated in Figure 8f. The charge–discharge profiles after 1500 and 4000 cycles are presented in Figure 8g,h. They show very good Coulombic efficiency (98%) after 1500 cycles, which further increased during cycling and attained almost 100% after 4000 cycles. These stabilities and Coulombic efficiency results highlight the good energy storage properties of the material with little interference of any parasitic reactions.

### Surface and Structural Characterization of Cycled Electrodes and Discussion

**Figure 9:**
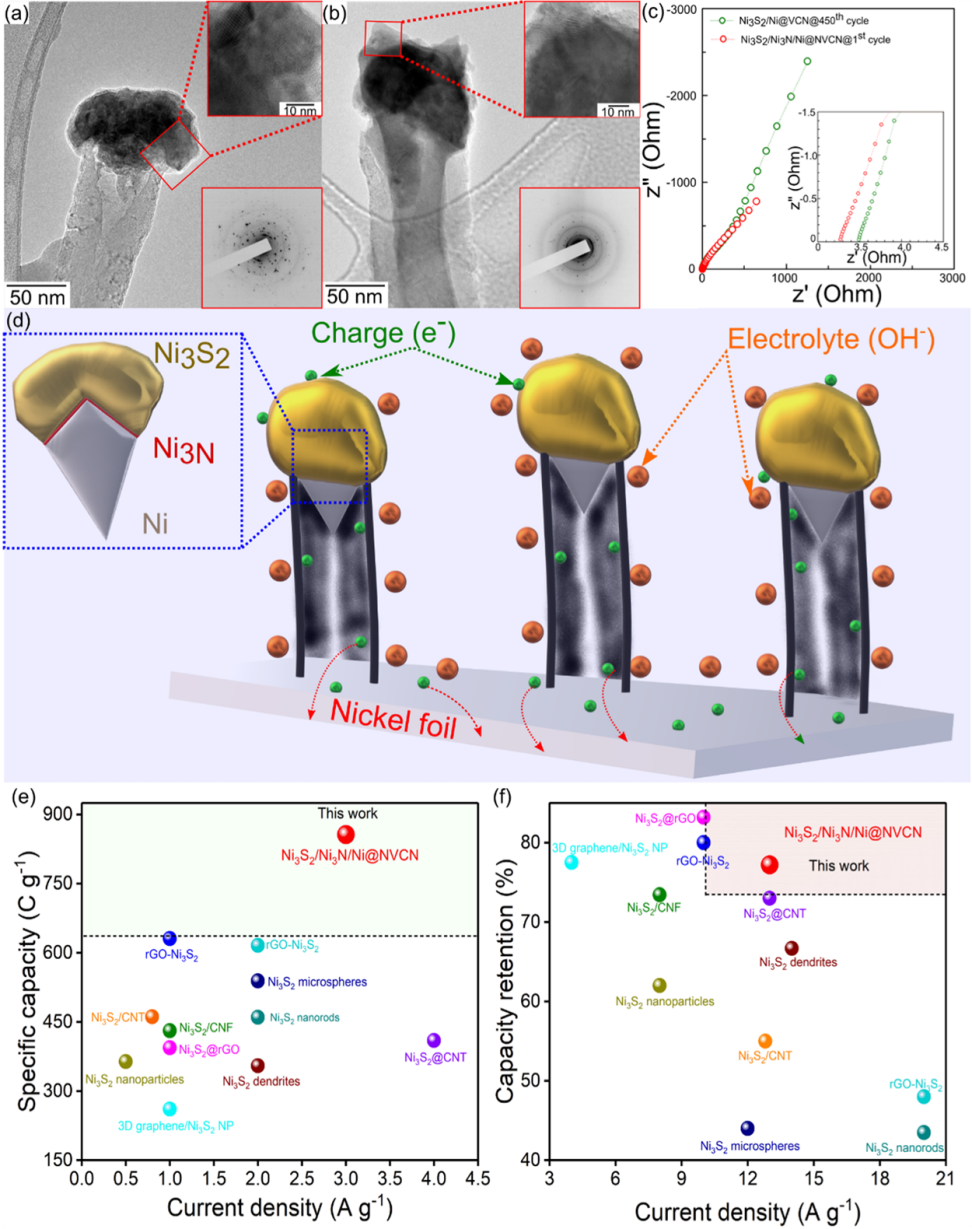
TEM and HR-TEM micrographs of (a) Ni_3_S_2_/Ni@VCN and (b) Ni_3_S_2_/Ni_3_N/Ni@NVCN electrodes after 4000 cycles, with the SAED pattern (inverted intensity), recorded over numerous particulates presented as the inset. (c) EIS comparison plots for Ni_3_S_2_/Ni@VCN after 450 cycles and the Ni_3_S_2_/Ni_3_N/Ni@NVCN electrode after the first cycle. (d) Schematic presentation of Ni_3_S_2_/Ni_3_N/Ni@NVCN as the electrode material for redox-based supercapacitors. (e) Comparison of specific capacity of Ni_3_S_2_-based electrodes with Ni_3_S_2_/Ni_3_N/Ni@NVCN. (f) Comparison of rate capability performances of Ni_3_S_2_-based electrodes with Ni_3_S_2_/Ni_3_N/Ni@NVCN.

The Ni_3_S_2_-based electrodes fabricated in this work exhibited excellent electrochemical performance for Faradaic supercapacitor applications, with a high specific capacity, and excellent rate capability and cycling stability. Of the two different electrodes studied, Ni_3_S_2_/Ni_3_N/Ni@NVCN showed ultra-high specific capacity (almost double) and outstanding rate capability. Excellent structural stability and the formation of structural defects could be considered important factors for high energy storage performance. Thus, the *ex situ* TEM analysis of both electrodes after 4000 cycles was performed, and the results are presented in Figure 9a,b. The vertical broccoli-like morphology was intact in both structures, and we did not observe any strain and no changes in the crystallinity or phase composition compared to the deposited structures before cycling. The SAED patterns of the electrodes after cycling are given in the insets. Additionally, a covering layer observed on both structures can be ascribed to the excellent intercalation with ions from the electrolyte, which seems dominant in Ni_3_S_2_/Ni_3_N/Ni@NVCN. HR-TEM images illustrate the formation of structural defects on both structures after electrochemical cycling. These structural defects can act as additional active sites for energy storage. Exposure to more active sites through the edges or structural vacancy defects can enhance the charge-storage mechanism of Ni_3_S_2_. The presence of carbon, sulfur, nitrogen, and nickel, along with potassium in the XPS spectra of the cycled electrodes, is the evidence of the interaction between the electrode material and the electrolyte during the electrochemical reaction (Figure S7). Another possible reason for the higher capacity achieved by the Ni_3_S_2_/Ni_3_N/Ni@NVCN electrode could be the improvement in the electrical conductivity of the structure and the change in internal resistance. To understand this property, EIS measurements were conducted and compared with those of a fresh electrode of Ni_3_S_2_/Ni_3_N/Ni@NVCN and Ni_3_S_2_/Ni@VCN after 450 cycles (Figure 9c). A magnified view of the Nyquist plot (Figure 9c) shows a lower ESR value for Ni_3_S_2_/Ni_3_N/Ni@NVCN. More importantly, the phase angle behavior of Ni_3_S_2_/Ni_3_N/Ni@NVCN in the mid–high-frequency range has a similar feature to Ni_3_S_2_/Ni@VCN after 450 cycles (Figure S8). This means that fresh Ni_3_S_2_/Ni_3_N/Ni@NVCN electrodes, even without any prior cycling, have lower diffusion limitations, and therefore, cycling is not needed to attain a stable electrochemical response. Further evidence comes from the GCD and rate capability data, which did not rise above 100% (Figure 8c,d). This could be ascribed to the higher hydrophilicity offered by Ni_3_S_2_/Ni_3_N/Ni@NVCN electrodes due to N-doping, which helped electrolyte wetting at all the active material sites from early cycling.

Based on all these findings, a possible mechanism of charge storage in Ni_3_S_2_/Ni_3_N/Ni@NVCN electrodes is presented in Figure 9d. The N-doped VCN backbone not only acts as a support for the controlled growth of Ni_3_S_2_ but also enhances pseudocapacitance performance. Additionally, the vertical alignment of the N-doped VCN backbone can serve as a conductive framework to provide an efficient pathway for rapid electron transport and possibly OH^–^ ion diffusion. The free-standing structure of the N-doped VCN and the capped structure of Ni_3_S_2_ provide a large surface area to interact with the electrolyte during electrochemical reactions. Intimate contact between the Ni_3_S_2_ active material and the current collector eases charge-transfer reactions and enhances rate capability and capacity.

The Ni_3_S_2_/Ni_3_N/Ni@NVCN electrode developed in this work exhibits superior specific capacity even at a higher current density compared to other Ni_3_S_2_-based electrodes (Figure 9e). In addition, the electrode possesses one of the best rate capability performances reported compared to other Ni_3_S_2_-based electrodes (Figure 9f). A comparison of the electrochemical performance of various Ni_3_S_2_-based electrodes and Ni_3_S_2_/Ni_3_N/Ni@NVCN is presented in Table S4, showing the potential of the material developed in this work for redox-based electrodes.

## Conclusions

In summary, we fabricated metal sulfide–carbon nanostructure-based hierarchical battery-type electrodes for Faradaic supercapacitor applications. One of the prepared hybrid binder-free electrodes consisted of trinickel disulfide (Ni_3_S_2_) on a single-crystal metallic Ni nanoparticle core terminated on VCNs in the form of Ni_3_S_2_/Ni@VCN. The other electrode consisted of the same components with a nickel nitride (Ni_3_N) interlayer in the form of Ni_3_S_2_/Ni_3_N/Ni@NVCN. VCN structures were directly deposited on a conductive Ni substrate using plasma deposition. Later, the deposited Ni@VCN structures were subjected to nitrogen plasma post-treatment to fabricate Ni_3_N/Ni@NVCN. In the last step, Ni@VCN and Ni_3_N/Ni@NVCN structures were thermally annealed at low temperature in the presence of H_2_S gas to form nickel sulfide (Ni_3_S_2_), which was polycrystalline in Ni@VCN and single crystalline in Ni_3_N/Ni@NVCN. Both structures possessed a unique broccoli-like hierarchical morphology.

The hybrid Ni_3_S_2_/Ni@VCN- and Ni_3_S_2_/Ni_3_N/Ni@NVCN-structured materials were tested as binder-free electrodes for energy storage applications in redox supercapacitors. Ni_3_S_2_/Ni@VCN electrodes delivered a capacity of 479 C g^–1^ with a good rate capability of 84% at 10 A g^–1^. The Ni_3_S_2_/Ni_3_N/Ni@NVCN electrode delivered almost twice the capacity (856.3 C g^–1^), one of the best values ever attained by Ni_3_S_2_-based electrodes. Additionally, this electrode possessed an outstanding rate capability by retaining 77.2% of initial capacity even at a higher current density of 13 A g^–1^. Both electrodes showed excellent cycling stability and retained above 80% initial capacity after 4000 cycles. The presence of nitrogen and defects in the VCN backbone, the hierarchical broccoli-like structure of nickel sulfide with a large surface area, the formation of additional defects in the active material during the electrochemical reaction, and the direct contact between the current collector and active material all contributed to enhanced electrochemical performance.

The electrode fabrication technique developed in this work is one of the fastest approaches for preparing Ni_3_S_2_-based binder-free electrodes and produced Faradaic electrodes with enhanced electrochemical performance. The ultra-high capacity and outstanding rate capability of the prepared Ni_3_S_2_/Ni_3_N/Ni@NVCN structures represent a step change over current battery-type electrodes, which typically display high capacity and low rate capability. This work, therefore, addresses one of the main challenges in battery-type electrodes for redox-based supercapacitors and suggests that Faradaic supercapacitor performance can be dramatically improved in the near future.

## Experimental Section

### Synthesis of the Carbon Nanostructure

Synthesis of carbon nanostructures was carried out by PECVD in a radio-frequency (RF) inductively coupled plasma system at low pressure. The RF generator (13.56 MHz) was coupled through a Nagoya type III inductive coil (14 cm long with a diameter of 8.5 cm) to a cross-shaped glass tube. The diameter of the tube was 8 cm with a length of 40 cm on each arm, and the reactor was air-cooled externally. Polycrystalline 25 μm thick nickel foil (thermally annealed 99% metal basis, Alfa Aesar) with dimensions 2 × 2 cm^2^ was used as the substrate for the synthesis of the carbon nanostructures. The Ni-foil substrate was placed directly in the plasma discharge region in the center of the coil using a stainless-steel substrate holder, which was ground during the growth process. Methane (CH_4_) at a flow rate of 20 sccm fed through a mass-flow controller was used as the carbon precursor. Before the experiment, the system was pumped down to 10 Pa with a rotary vacuum pump, and the total pressure of the system was kept at ∼30 Pa during the experiment. Depositions were performed for 4 min at a plasma power of 800 W to synthesize the carbon nanostructures on the Ni foil. The substrate temperature reached approximately 750–800 °C due to plasma heating effects.

### Fabrication of N-Carbon Nanostructures

N-doped carbon nanostructures were fabricated using another RF inductively coupled plasma system at low pressure, as described in the literature.^64^ The plasma system consisted of an 80 cm long horizontal glass tube with a diameter of 4 cm. The RF generator was inductively coupled to the system using a nine-turn water-cooled inductive coil. Nitrogen (N_2_) was used as the dopant gas at a flow rate of 100 sccm. The system was pumped down to 1 Pa before the experiments using a two-stage rotary pump and maintained at 30 Pa during the experiments. The sample was placed 10 cm away from the center of the glow discharge into the post-glow region. Plasma was generated at an RF power of 300 W. Plasma post-treatment was carried out using an incremental method, where the plasma was switched off during each treatment periodically to keep the surface temperature minimized. This was done in three steps of 10 s (total 30 s) and equal cooling periods.

### Synthesis of Nickel Sulfide

Nickel sulfide was formed on top of the nickel nanoparticle, which resided on the carbon nanostructure and was covered by nickel nitride. The nickel sulfide part of the composite was synthesized in an 80 cm long quartz tube furnace with a 45 cm heating zone (OTF-1200X-II, MTI Corp.). The carbon nanostructures and N-doped carbon nanostructures deposited on Ni foil were placed in the middle of the tube on a glass substrate. The tube was pumped-down with a rotary pump to <1 Pa before the experiment and then filled with H_2_S gas to a pressure close to atmospheric pressure. The temperature of the chamber was elevated to 125 °C at a rate of 6 °C/min, and the samples were annealed for 3 h. Afterward, the furnace was left to cool down to room temperature, and H_2_S gas was pumped out.

### Characterization Techniques

Surface morphology of the samples was explored by a scanning electron microscope [Prisma E scanning electron microscope, Thermo Fisher Scientific Inc.], operated at 5 kV. The crystal structure and phase composition of the samples were analyzed with a transmission electron microscope (JEM-2100, Jeol Inc.) operating at 200 kV and additionally equipped with an energy-dispersive X-ray spectrometer (EX-24063JGT, Jeol Inc.). Micrographs were recorded by a slow-scan CCD camera (Orius SC1000, Gatan). Structural properties of the prepared structures were investigated by Raman spectra recorded using an NTEGRA confocal Raman spectrometer at an excitation wavelength of 488 nm. The Raman spectra were recorded at four different spots on the samples. XPS (PHI-TFA XPS spectrometer, Physical Electronics Inc) analysis was employed to evaluate the surface composition and bonding environment of the samples using an Al-monochromatic X-ray source at an energy of 1486.6 eV.

### Electrochemical Measurements

Nickel sulfide/carbon nanostructures directly grown on Ni foil were used as binder-free working electrodes without using any conductive carbon and a polymeric binder. All the samples were subjected to electrochemical measurements without any further treatment. Electrochemical measurements were performed in a three-electrode setup using Pt as a counter electrode and saturated calomel as a reference electrode. CV was scanned in the potential window from −0.2 to 0.45 V and GCD from −0.1 to 0.4 V in a freshly prepared 2 M KOH electrolyte. EIS was conducted at an open-circuit potential (OCP) in the frequency range of 10^5^–10^–2^ Hz at an RMS amplitude of 10 mV. Gamry interface 5000E was used for all the measurements.

The power-law equation used for analyzing the diffusional processes from CV data is given by
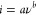
3where *i* is the current (mA cm^–2^), *a* and *b* are the variable parameters, and ν is the scan rate (mV s^–1^).

The specific capacity of the electrodes was calculated using the equation^55,70^
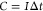
4where *C* is the specific capacity (C g^–1^; the values are also presented in mA h g^–1^), *I* is the current density (A g^–1^), and Δ*t* is the discharge time (s).

## Supporting Information Available

SEM micrograph of VCN and NVCN; TEM micrograph of NVCN; EDS elemental mapping of the individual Ni_3_S_2_/Ni@VCN structure; SAED patterns of Ni_3_S_2_/Ni@VCN and Ni_3_S_2_/Ni_3_N/Ni@NVCN; XPS survey spectra of nanostructures; XPS deconvolution details of Ni_3_S_2_/Ni_3_N/Ni@NVCN ; water contact angle measurements of Ni_3_S_2_/Ni@VCN and Ni_3_S_2_/Ni_3_N/Ni@NVCN electrodes; XPS survey spectra of cycled electrodes; comparison of the specific capacity and rate capabilities of Ni_3_S_2_-based electrodes; and EIS Bode plot comparison of electrodes (PDF)
