# Supplementary material for: Advanced Carbon–Nickel Sulfide Hybrid Nanostructures: Extending the Limits of Battery-Type Electrodes for Redox-Based Supercapacitor Applications
Source: ACS Appl Mater Interfaces. 2021 Apr 21;13(17):20559–72. doi: 10.1021/acsami.1c03053 (PMC8289178; doi:10.1021/acsami.1c03053)
Supplement: Supplementary file 1 — am1c03053_si_001.pdf [file am1c03053_si_001.pdf]

Supporting Information

Advanced Carbon-Nickel Sulfide Hybrid Nanostructures: Extending the Limits of Battery-Type Electrodes for Redox-Based Supercapacitor Applications

Neelakandan M Santhosh[1,2], Kush K. Upadhyay[3,4], Petra Stražar[1,2], Gregor Filipič[1], Janez Zavašnik[1], André Mão de Ferro[3], Rui Pedro Silva[3], Elena Tatarova[5], Maria de Fátima Montemor[4], Uroš Cvelbar[1,2*]*

[1]Department of Gaseous Electronics, Jožef Stefan Institute, Jamova cesta 39, SI-1000 Ljubljana, Slovenia

[2]Jožef Stefan International Postgraduate School, Jamova cesta 39, SI-1000 Ljubljana, Slovenia

[3]Charge2C-NewCap, Av. José Francisco Guerreiro, nº28 Paiã Park, Armazém A2.12, 1675-078 Pontinha – Odivelas, Portugal

[4]Centro de Química Estrutural-CQE, Departamento de Engenharia Química, Instituto Superior Técnico, Universidade de Lisboa,1049-001 Lisboa, Portugal.

[5]Instituto de Plasmas e Fusão Nuclear, Instituto Superior Técnico, Universidade de Lisboa, Lisboa-1049, Portugal.

Corresponding Authors

Uroš Cvelbar *- Department of Gaseous Electronics, Jožef Stefan Institute, Jamova cesta 39, SI-1000 Ljubljana, Slovenia; Jožef Stefan International Postgraduate School, Jamova cesta 39, SI-1000 Ljubljana, Slovenia. Email: uros.cvelbar@ijs.si*

Kush K. Upadhyay *- Charge2C-NewCap, Av. José Francisco Guerreiro, nº28 Paiã Park, Armazém A2.12, 1675-078 Pontinha – Odivelas, Portugal; Centro de Química Estrutural-CQE, Departamento de Engenharia Química, Instituto Superior Técnico, Universidade de Lisboa,1049-001 Lisboa, Portugal. Email: kush.upadhyay@c2cnewcap.com*

Vertically aligned carbon nanotube (VCN) structures with an approximate height of ~1 μm were used for N-doping and sulfur treatment. Vertical alignment and uniform growth of VCN structures are shown in **Figure S1a**. There was no structural damage observed after N-doping and the surface morphology after N-doping is presented in **Figure S1b**.

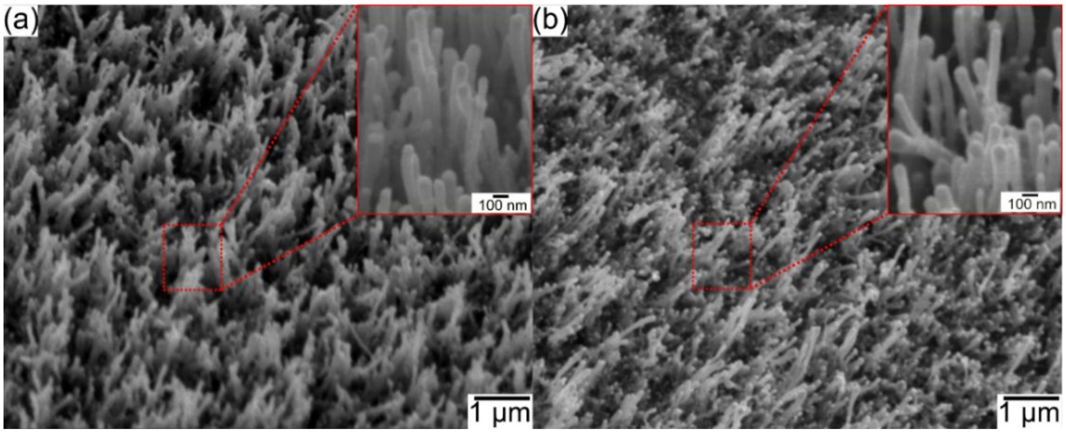

Figure S1. SEM secondary electrons (SE) micrograph of vertical carbon nanostructures: (a) VCN before N-doping and (b) VCN after N-doping.

After N-doping, there were no detectable changes observed in the morphology of the VCN structures. However, by HR-TEM observation of the terminal Ni monocrystal, a nitride layer is observed around the exposed part of the Ni in N-VCN. The Ni-nitride phase forms as an epitaxial layer with a thickness of ~5 nm on all free Ni surfaces, while not on those protected, even partially, by carbon layers from VCN. From the FFT of the HR-TEM image, the phase was determined to be Ni_3N, epitaxially grown on the single-crystal Ni (**Figure S2**).

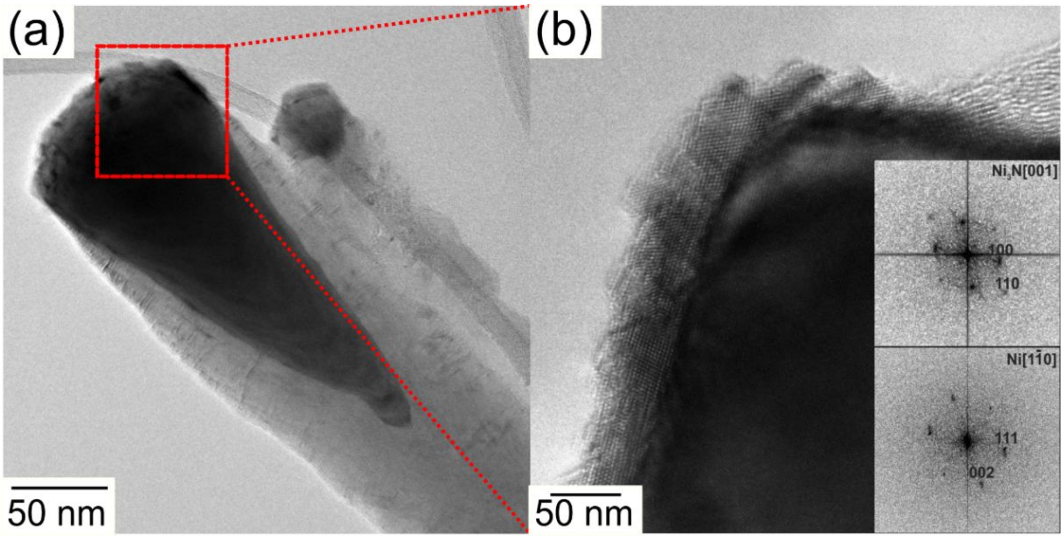

Figure S2. (a) TEM micrograph of N-doped VCN, capped by faceted Ni monocrystal covered by Ni_3N, (b) HR-TEM micrograph of epitaxial Ni_3N layer with a thickness of ~5 nm, grown atop of the single-crystal Ni. FFT patterns in the inset are indexed for Ni_3N (surface layer) and Ni (bulk).

The elemental analysis of the individual Ni_3S_2/Ni@VCN structure has been done by EDX. It is evident that after the sulfur treatment, the sulfur neither damaged the vertical alignment, incorporated itself into the carbon backbone, nor deposited as elemental S on the nanostructure. Processes have only made significant changes at the Ni monocrystal terminal (**Figure S3**).

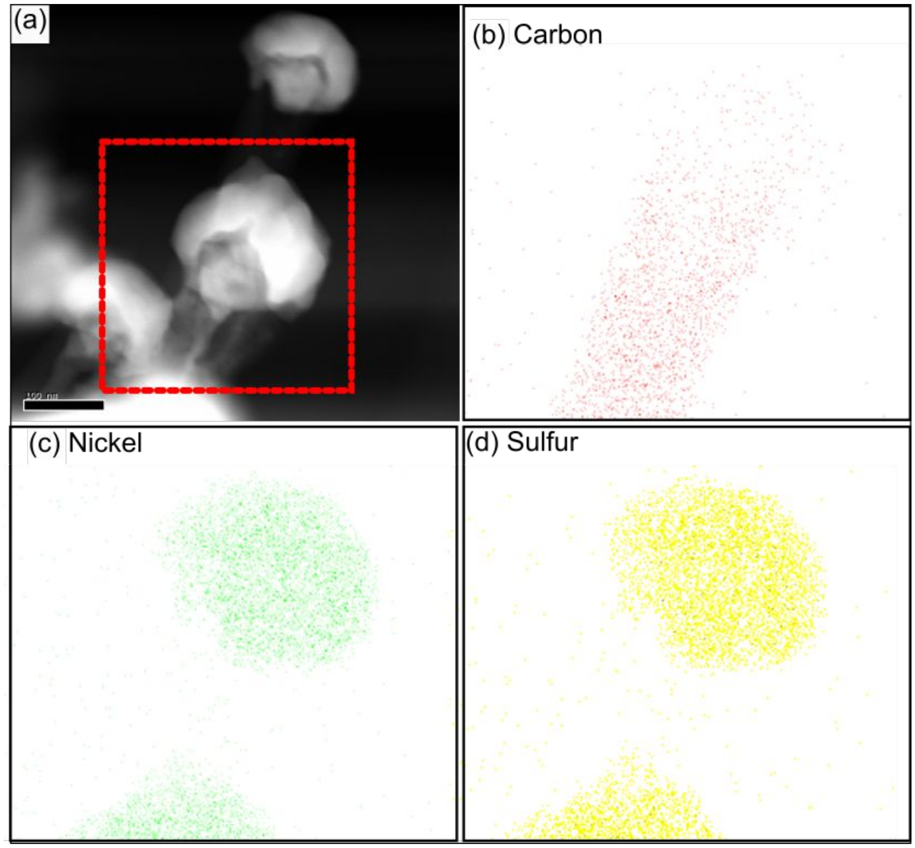

Figure S3. a) HAADF-STEM; corresponding EDS elemental mapping of individual Ni_3S_2/Ni@VCN structure b-d).

The SAED patterns of nickel sulfides confirm that the fabricated nickel sulfides possess the Ni_3S_2 phase (Heazlewoodite). The diffraction from the Ni_3N layer is also observed from N-doped VCN structures confirms that Ni_3N is protected even after the sulfur treatment. Experimental and simulated SAED pattern for Ni_3S_2, Ni_3N, Ni and VCN is presented in **Figure S4**.

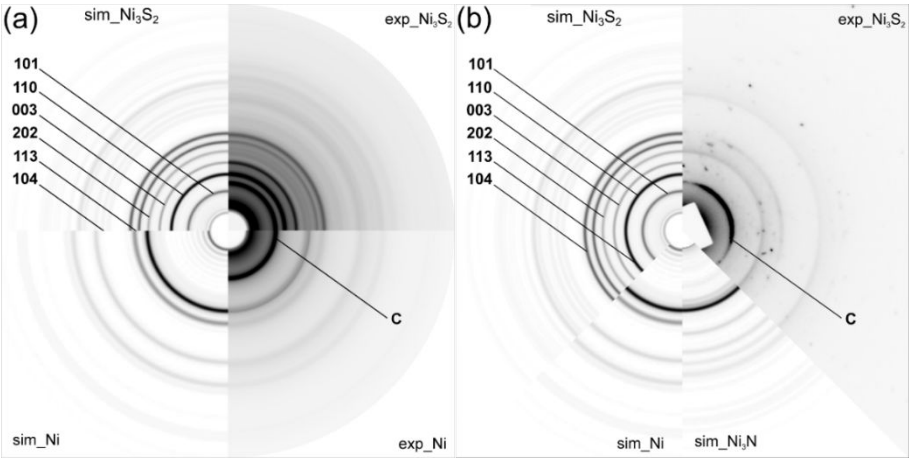

Figure S4. Phase composition analysis of nickel sulfides: (a) experimental SAED pattern for Ni_3S_2, Ni, and VCN, and corresponding simulated patterns for Ni_3S_2, Ni, and C in Ni_3S_2/Ni@VCN; (b) experimental SAED pattern for Ni_3S_2, Ni_3N, Ni, and VCN, and corresponding simulated patterns for Ni_3S_2, Ni, Ni_3N, and C in Ni_3S_2/ Ni_3N/Ni@NVCN.

Surface components and the chemical composition of the prepared nanostructures were characterized by XPS analysis. Peaks at 284.6, 531.8, 850.2, and 162.2 eV confirm the presence of C, O, Ni, and S in the nanostructure as presented in **Figure S5**. A peak around 400 eV confirms the presence of nitrogen in the Ni_3S_2/ Ni_3N/Ni@NVCN structures.

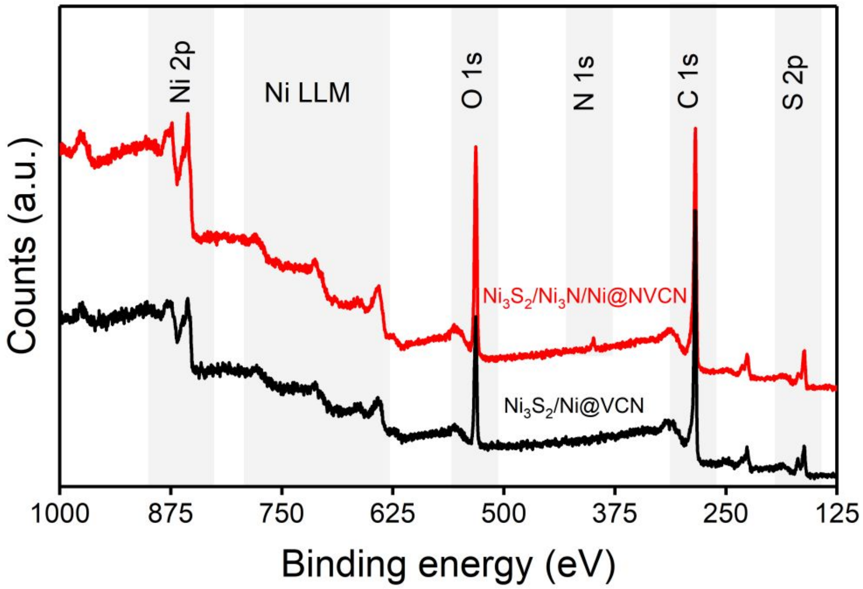

Figure S5. XPS survey spectra of the prepared nanostructures and elemental analysis.

Detailed information on the peak position and area of the deconvoluted peaks in the high-resolution XPS data of the carbon, sulfur, and nickel peaks of the samples are described in Tables S1, S2, and S3. The high-resolution XPS peaks were deconvoluted using the Gauss–Lorentz method considering Shirley-type background deduction.

Table S1. Peak position, FWHM, and area under the curve of the peak components in C 1s spectra of Ni_3S_2/Ni@VCN and Ni_3S_2/ Ni_3N/Ni@NVCN.

Deconvoluted peaks	Ni_3S_2/Ni@VCN			Ni_3S_2/ Ni_3N/Ni@NVCN		
	Position (eV)	FWHM (eV)	Area %	Position (eV)	FWHM (eV)	Area %
Peak 1	283.5	1	3.7	283.5	1	3.1
Peak 2	284.6	1	59.5	284.6	1	58.2
Peak 3	285.4	1.2	20.7	285.4	1.2	23.4
Peak 4	286.7	1.5	8.0	286.2	1.5	4.5
Peak 5	288.2	1.8	4.8	286.7	1.5	3.8
Peak 6	290.5	2.0	3.0	288.2	1.8	4.1
Peak 7				290.5	2.0	2.8

Table S2. Peak position, FWHM, and roughly estimated concentration of the peak components in S 2p spectra of Ni_3S_2/Ni@VCN and Ni_3S_2/ Ni_3N/Ni@NVCN.

Deconvoluted peaks	Ni_3S_2/Ni@VCN			Ni_3S_2/ Ni_3N/Ni@NVCN		
	Position (eV)	FWHM (eV)	Area %	Position (eV)	FWHM (eV)	Area %
Peak 1	161.7	1.2	24.6	161.7	1.2	25.9
Peak 2	163.0	1.9	37.1	163.0	2.0	45.9
Peak 3	165.4	2.5	12.3	165.4	2.5	9.0
Peak 4	169.0	2.2	25.8	169.0	2.4	19.1

Table S3. Peak position, FWHM, and area under the curve of the peak components in Ni 2p spectra of Ni_3S_2/Ni@VCN and Ni_3S_2/ Ni_3N/Ni@NVCN.

Deconvoluted peaks	Ni@VCNs			Ni_3S_2/Ni@VCNs		
	Position (eV)	FWHM (eV)	Area %	Position (eV)	FWHM (eV)	Area %
Peak 1	853.4	1	27.7	853.4	1.3	18.1
Peak 2	856.2	2.5	40.4	856.2	2.8	55.5
Peak 3	861.0	5.4	31.7	861.0	5.0	26.2

Both the Ni_3S_2/Ni@VCN and Ni_3S_2/ Ni_3N/Ni@NVCN electrodes were shown hydrophobic nature during the electrochemical reaction. However, Ni_3S_2/Ni@VCN electrodes' characteristics were more hydrophobic. Thus, contact angle measurements were performed to analyze the surface properties. Ni_3S_2/Ni@VCN electrodes (125±3°) exhibit a more hydrophobic nature than the Ni_3S_2/ Ni_3N/Ni@NVCN electrodes (108±5°), which indicates that the affinity towards the water was increased after the nitrogen plasma treatment. Hence, the treatment resulted in improved electrochemical performance.

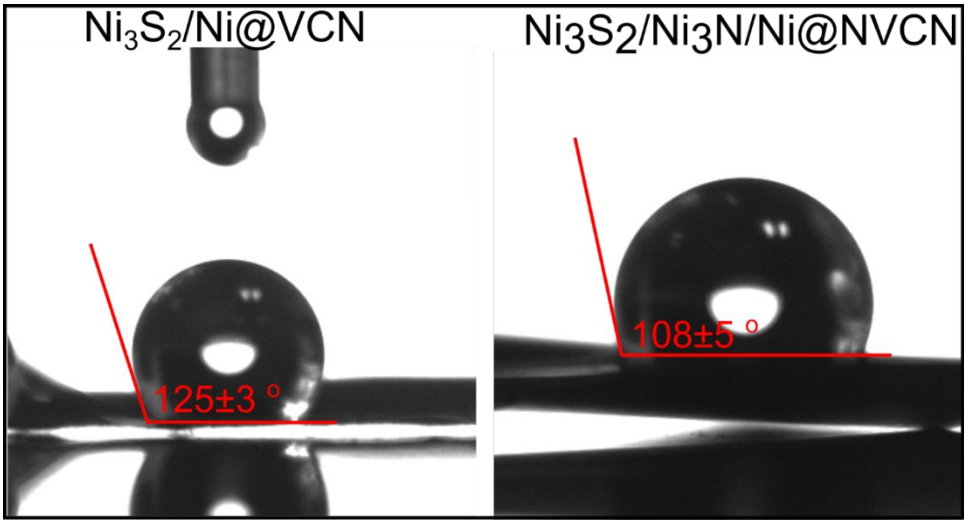

Figure S6. Water contact angle measurements of Ni_3S_2/Ni@VCN and Ni_3S_2/ Ni_3N/Ni@NVCN electrodes.

Electrodes were characterized by XPS analysis again after 4000 cycles to investigate the loss of elements after cycling. XPS survey spectra of the cycled electrodes are shown in **Figure S7**. Along with the electrode elements, a small peak in the spectra corresponding to the presence of potassium could be a result of excellent intercalation of electrode materials with the electrolyte. However, there is no structural damage or phase change observed from the TEM images, suggesting excellent chemical stability of the electrode material.

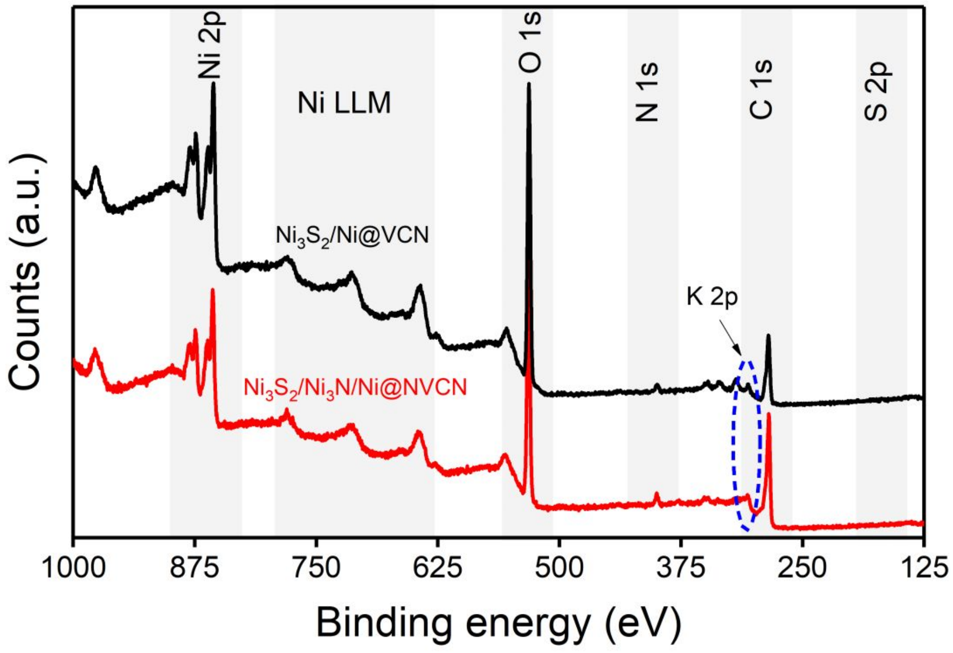

Figure S7. XPS survey spectra of the cycled electrodes and elemental analysis.

Phase angle behavior of Ni_3S_2/Ni_3N/Ni@NVCN after 1st cycle and Ni_3S_2/Ni@VCN after 450th cycle (after electrochemical stabilization) is presented in Figure S8.

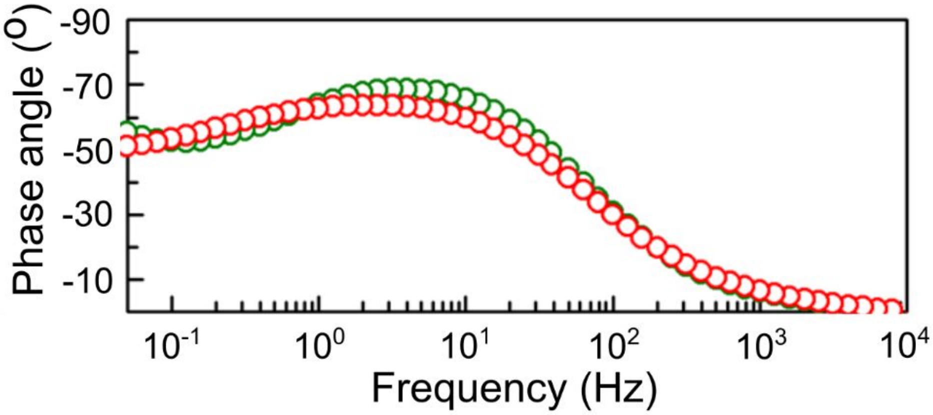

Figure S8. EIS Bode plot comparison of Ni_3S_2/Ni@VCN after 450th cycle (green) and Ni_3S_2/Ni_3N/Ni@NVCN electrode after 1st cycle (red).

Ni_3S_2/Ni_3N/Ni@NVCN electrode has a similar feature to Ni_3S_2/Ni@VCN after 450 cycles in the mid-high frequency range. This indicates that fresh Ni_3S_2/Ni_3N/Ni@NVCN electrodes, even

without any prior cycling, have lower diffusion limitations, and therefore cycling is not needed to attain a stable electrochemical response.

A comparison of the supercapacitor performances of Ni3S2-based battery-type electrodes has presented in Table S4. The comparison confirms that the electrode fabricated in this work, $Ni_3S_2/Ni_3N/Ni$@NVCN-based electrodes, has shown one of the best specific capacity and rate capability among the other varieties.

Table S4. Comparison of the specific capacity and rate capabilities of Ni_3S_2-based electrodes.

Electrode	Voltage (V)	Specific capacity (C g^{-1})	Current density (A g^{-1})	Capacity retention (%) @ current density (A g^{-1})	Number of cycles @ current density (A g^{-1})	Retention (%)	Ref
Ni_3S_2@CNT	-0.15-0.55	410 (586 F g^{-1})	4	73@13	1500@5.3	88	[1]
Ni_3S_2@rGO	0.1-0.55	394 (875 F g^{-1})	1	83.2@10	1000@5	91.3	[2]
Ni_3S_2 dendrites	0-0.5	355 (710 F g^{-1})	2	66.7@14	2000@5	100	[3]
Ni_3S_2 microspheres	-0.1-0.45	539 (982 F g^{-1})	2	44@12	1000@2	96.9	[4]
Ni_3S_2 nanoparticles	0-0.4	364 (911 F g^{-1})	0.5	62@8	1000@0.5	90	[5]
rGO-Ni_3S_2	0-0.48	631 (1315 F g^{-1})	1	80@10	1000@1	115	[6]
Ni_3S_2/CNT	0-0.45	461 (1024 F g^{-1})	0.8	46.24@25.6 (55@12.8)	1000@3.2	80	[7]
Ni_3S_2/CNF	0-0.45	431 (957 F g^{-1})	1	73.45@8	1000@4	83.5	[8]
3D graphene/Ni_3S_2 NP	1.15-1.55	261 (652.5 F g^{-1})	1	77.5@4	2000@1	93	[9]
3D porous graphene/Ni_3S_2	0-0.75	444.7 (593 F g^{-1})	0.5	33.7@50	5000@20	92.3	[10]
rGO-Ni_3S_2	0-0.45	616 (1368 F g^{-1})	1	48@20	5000@5	92.7	[11]
Ni_3S_2/Ni	0-0.45	581.85 (1293 F g^{-1})	5 mA cm^{-2}	52.82@25 mA cm^{-2}	1000@25 mA cm^{-2}	69	[12]
Ni_3S_2 nanorods	0-0.45	460 (1022.2 F g^{-1})	2	43.48@20	3000@2.4	93.1	[13]
Ni_3S_2 nanowire	0-0.5	5.8 F cm^{-2}	5 mA cm^{-2}	50@30 mA cm^{-2}	5000@70 mA cm^{-2}	83.7	[14]
Ni_3S_2@Ni	0-0.5	1980 mF cm^{-2}	5 mA cm^{-2}	68.5@20 mA cm^{-2}	4000@20 mA cm^{-2}	100	[15]
Ni_3S_2@Ni	0-0.5	845 (1691 F g^{-1})	1.43	56.91@17.15	5000@10 mA cm^{-2}	106	[16]
Ni_3S_2	0-0.55	3.42 F cm^{-2}	1 mA cm^{-2}	45.32@10 mA cm^{-2}	4250@7 mA cm^{-2}	102	[17]
Ni_3S_2@Ni3N/Ni@VCN	-0.1-0.4	856.3	3	77.2@13	4000@13	83	**This Work**

References

1. Zhu, T.; Wu, H. Bin; Wang, Y.; Xu, R.; Lou, X. W. Formation of 1D hierarchical structures composed of Ni3S 2 nanosheets on CNTs backbone for supercapacitors and photocatalytic H2 production. Adv. Energy Mater. 2012, doi:10.1002/aenm.201200269.

2. Ou, X.; Luo, Z. One-step synthesis of Ni3S2 nanoplatelets on graphene for high performance supercapacitors. RSC Adv. 2016, doi:10.1039/c5ra22426h.
3. Zhang, Z.; Huang, Z.; Ren, L.; Shen, Y.; Qi, X.; Zhong, J. One-pot synthesis of hierarchically nanostructured Ni3S2 dendrites as active materials for supercapacitors. Electrochim. Acta 2014, 149, 316–323, doi:10.1016/j.electacta.2014.10.097.
4. Li, G.; Cong, Y.; Zhang, C.; Tao, H.; Sun, Y.; Wang, Y. Hierarchical nanosheet-based Ni3S2 microspheres grown on Ni foam for high-performance all-solid-state asymmetric supercapacitors. Nanotechnology 2017, doi:10.1088/1361-6528/aa829d.
5. Li, J. J.; Hu, Y. X.; Liu, M. C.; Kong, L. Bin; Hu, Y. M.; Han, W.; Luo, Y. C.; Kang, L. Mechanical alloying synthesis of Ni3S2 nanoparticles as electrode material for pseudocapacitor with excellent performances. J. Alloys Compd. 2016, doi:10.1016/j.jallcom.2015.09.221.
6. Lin, H.; Liu, F.; Wang, X.; Ai, Y.; Yao, Z.; Chu, L.; Han, S.; Zhuang, X. Graphene-Coupled Flower-Like Ni3S2 for a Free-Standing 3D Aerogel with an Ultra-High Electrochemical Capacity. Electrochim. Acta 2016, doi:10.1016/j.electacta.2016.01.064.
7. Dai, C. S.; Chien, P. Y.; Lin, J. Y.; Chou, S. W.; Wu, W. K.; Li, P. H.; Wu, K. Y.; Lin, T. W. Hierarchically structured Ni3S2/carbon nanotube composites as high performance cathode materials for asymmetric supercapacitors. ACS Appl. Mater. Interfaces 2013, 5, 12168–12174, doi:10.1021/am404196s.
8. Yu, W.; Lin, W.; Shao, X.; Hu, Z.; Li, R.; Yuan, D. High performance supercapacitor based on Ni3S2/carbon nanofibers and carbon nanofibers electrodes derived from bacterial cellulose. J. Power Sources 2014, doi:10.1016/j.jpowsour.2014.08.064.
9. Li, Z.; Li, B.; Liao, C.; Liu, Z.; Li, D.; Wang, H.; Li, Q. One-pot construction of 3-D graphene nanosheets/Ni3S2 nanoparticles composite for high-performance supercapacitors. Electrochim. Acta 2017, doi:10.1016/j.electacta.2017.09.070.
10. Wu, P.; Wang, D.; Ning, J.; Zhang, J.; Feng, X.; Dong, J.; Hao, Y. Novel 3D porous graphene/Ni3S2 nanostructures for high-performance supercapacitor electrodes. J. Alloys Compd. 2018, doi:10.1016/j.jallcom.2017.10.060.
11. Namdarian, A.; Tabrizi, A. G.; Maseleno, A.; Mohammadi, A.; Moosavifard, S. E. One step synthesis of rGO-Ni3S2 nano-cubes composite for high-performance supercapacitor electrodes. Int. J. Hydrogen Energy 2018, doi:10.1016/j.ijhydene.2018.07.178.
12. Krishnamoorthy, K.; Veerasubramani, G. K.; Radhakrishnan, S.; Kim, S. J. One pot hydrothermal growth of hierarchical nanostructured Ni3S2 on Ni foam for supercapacitor application. Chem. Eng. J. 2014, doi:10.1016/j.cej.2014.04.006.
13. Wen, J.; Li, S.; Zhou, K.; Song, Z.; Li, B.; Chen, Z.; Chen, T.; Guo, Y.; Fang, G. Flexible coaxial-type fiber solid-state asymmetrical supercapacitor based on Ni 3 S 2 nanorod array and pen ink electrodes. J. Power Sources 2016, 324, 325–333, doi:10.1016/j.jpowsour.2016.05.087.
14. Ma, J.; Li, W.; Zhang, X.; Cheng, Y.; Zhang, F. Free-standing Ni3S2 nanowire derived from in-situ synthetized coordination supramolecular as electrode materials for high

performance asymmetric supercapacitors. Appl. Surf. Sci. 2020, doi:10.1016/j.apsusc.2019.145074.
15. Shen, M.; Liu, J.; Liu, T.; Yang, C.; He, Y.; Li, Z.; Li, J.; Qian, D. Oxidant-assisted direct-sulfidization of nickel foam toward a self-supported hierarchical Ni3S2@Ni electrode for asymmetric all-solid-state supercapacitors. J. Power Sources 2020, doi:10.1016/j.jpowsour.2019.227408.
16. Chen, S.; Li, Y.; Wu, B.; Wu, Z.; Li, F.; Wu, J.; Liu, P.; Li, H. 3D meso/macroporous Ni3S2@Ni composite electrode for high-performance supercapacitor. Electrochim. Acta 2018, 275, 40–49, doi:10.1016/j.electacta.2018.04.152.
17. Li, J.; Wang, S.; Xiao, T.; Tan, X.; Xiang, P.; Jiang, L.; Deng, C.; Li, W.; Li, M. Controllable preparation of nanoporous Ni 3 S 2 films by sulfuration of nickel foam as promising asymmetric supercapacitor electrodes. Appl. Surf. Sci. 2017, doi:10.1016/j.apsusc.2017.05.206.
